# Genetic predictors of lifelong medication-use patterns in cardiometabolic diseases

**DOI:** 10.1038/s41591-022-02122-5

**Published:** 2023-01-18

**Authors:** Tuomo Kiiskinen, Pyry Helkkula, Kristi Krebs, Juha Karjalainen, Elmo Saarentaus, Nina Mars, Arto Lehisto, Wei Zhou, Mattia Cordioli, Sakari Jukarainen, Joel T. Rämö, Juha Mehtonen, Kumar Veerapen, Markus Räsänen, Sanni Ruotsalainen, Mutaamba Maasha, FinnGen, Teemu Niiranen, Tiinamaija Tuomi, Veikko Salomaa, Mitja Kurki, Matti Pirinen, Aarno Palotie, Mark Daly, Andrea Ganna, Aki S. Havulinna, Lili Milani, Samuli Ripatti

**Affiliations:** 1 Institute for Molecular Medicine Finland (FIMM), University of Helsinki, Helsinki, Finland; 2 Finnish Institute for Health and Welfare, Helsinki, Finland; 3 Broad Institute of the Massachusetts Institute of Technology and Harvard University, Cambridge, MA USA; 4 Estonian Genome Center, Institute of Genomics, University of Tartu, Tartu, Estonia; 5 Analytic and Translational Genetics Unit, Massachusetts General Hospital, Boston, MA USA; 6 Department of Otorhinolaryngology – Head and Neck Surgery, University of Helsinki and Helsinki University Hospital, Helsinki, Finland; 7 Wihuri Research Institute, University of Helsinki, Helsinki, Finland; 8 Department of Internal Medicine, University of Turku, Turku, Finland; 9 Division of Medicine, Turku University Hospital, Turku, Finland; 10 Research Programs Unit, Clinical and Molecular Metabolism, University of Helsinki, Helsinki, Finland; 11 Lund University Diabetes Center, Malmo, Sweden; 12 Folkhälsan Research Centre, Helsinki, Finland; 13 Department of Endocrinology, Helsinki University Hospital, Helsinki, Finland; 14 Department of Public Health, Clinicum, Faculty of Medicine, University of Helsinki, Helsinki, Finland; 15 Department of Mathematics and Statistics, University of Helsinki, Helsinki, Finland; 16 Program in Medical and Population Genetics and Stanley Center for Psychiatric Research, Broad Institute of Harvard and MIT, Cambridge, MA USA

**Keywords:** Risk factors, Pharmacogenomics, Ischaemia

## Abstract

Little is known about the genetic determinants of medication use in preventing cardiometabolic diseases. Using the Finnish nationwide drug purchase registry with follow-up since 1995, we performed genome-wide association analyses of longitudinal patterns of medication use in hyperlipidemia, hypertension and type 2 diabetes in up to 193,933 individuals (55% women) in the FinnGen study. In meta-analyses of up to 567,671 individuals combining FinnGen with the Estonian Biobank and the UK Biobank, we discovered 333 independent loci (*P* < 5 × 10^–9^) associated with medication use. Fine-mapping revealed 494 95% credible sets associated with the total number of medication purchases, changes in medication combinations or treatment discontinuation, including 46 credible sets in 40 loci not associated with the underlying treatment targets. The polygenic risk scores (PRS) for cardiometabolic risk factors were strongly associated with the medication-use behavior. A medication-use enhanced multitrait PRS for coronary artery disease matched the performance of a risk factor-based multitrait coronary artery disease PRS in an independent sample (UK Biobank, *n* = 343,676). In summary, we demonstrate medication-based strategies for identifying cardiometabolic risk loci and provide genome-wide tools for preventing cardiovascular diseases.

## Abstract (web summary)

A new analysis of large biobanks uncovers genetic variants associated with longitudinal changes in medication for cardiometabolic diseases and presents polygenic scores of medication-use behavior.

## Main

Cardiovascular disease (CVD) is the leading cause of excess mortality in the developed countries^1^, and although approximately half of the variability in cardiometabolic diseases is heritable^2^, most related harm is preventable^3,4^. Pharmacotherapies targeting cardiometabolic risk factors—type 2 diabetes (T2D), hyperlipidemia and hypertension—remain at the core of CVD prevention^5,6^.

Challenges in pharmacological prevention of CVD involve identifying patients in need of therapy, setting the targets of the treatment and selecting therapies of adequate efficacy and acceptable risk profiles. In addition to socioeconomic factors, both the set and dose of medicines that patients start their treatment with and continue to use depends on factors such as cardiovascular risk profiles, disease etiology, drug responsiveness and adverse effects^5,6^. Abandoning or inadequately adhering to therapies worsens outcomes^7–10^. With limited tools to predict treatment suboptimality, pharmacotherapy is traditionally optimized in a reactive trial-and-error manner when patients experience side effects, miss their treatment targets or experience events such as myocardial infarction or stroke^5,6^. Real-world data from electronic health records and registries provide massive datasets with sufficient statistical power to explore long-term medication use. Behavioral patterns derived from prescription data can be used as a proxy to detect suboptimal or harmful prescriptions^11,12^. Such studies are essential to identify factors that influence interindividual variability in treatment response and can inform clinicians on initial treatments that are different from first-line therapies.

Genetic information has been proposed as a tool to optimize pharmacotherapy^13,14^ and advance drug development^15^. Despite the progress in disease genetics, we have relatively limited knowledge on the role of genetic factors driving the variation in lifelong patterns of medication use in cardiometabolic diseases. Meanwhile, large-scale genome-wide association studies (GWAS) have identified a complex polygenic architecture comprising hundreds of associated loci for lipid levels^16–18^, blood pressure^19,20^ and T2D^21,22^, and using genetic information in clinical risk prediction shows promise of clinical relevance^23,24^. A study of self-reported medication use in the UK Biobank (UKBB) revealed variants associated with medication use, but the self-reported nature and ambiguous names of medications may have limitations in the accuracy of the investigated phenotypes^25^. In contrast, pharmacogenetic associations, such as the *SLCO1B1* polymorphism (rs4149056) increasing risk for simvastatin-induced myopathy^26^, have been identified in smaller pharmacogenetic studies^27–29^, mostly having a relatively narrow focus on drug response as the primary outcome.

**Fig. 1:**
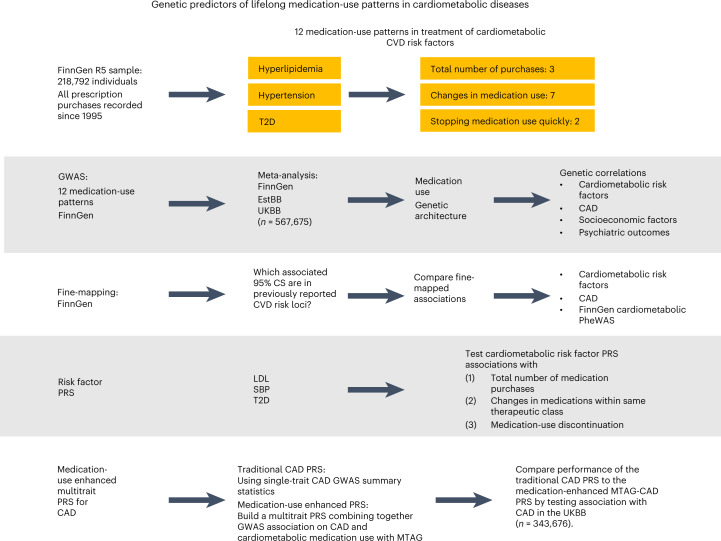
Workflow of the study. GWAS were performed for 12 phenotypes of medication-use patterns in treating hyperlipidemia, hypertension and T2D (three continuous analyses of the total number of medication purchases and nine binary analyses of medication changing and fast discontinuation) in FinnGen, with data capturing all medication purchases since 1 January 1995. Meta-analyses of up to 567,671 participants combined data in FinnGen (*n* = 29,990–193,933), EstBB (*n* = 5,110–184,892) and UKBB (*n* = 188,846). Fine-mapping was performed in FinnGen for all associated (*P* < 5 × 10^−8^) regions. Genetic architectures between medication-use traits and the underlying cardiometabolic risk were juxtaposed by comparing genome-wide significant associations, calculating LDSC regression genetic correlations and testing associations between PRS for LDL, SBP and T2D and the medication-use phenotypes. A medication-use enhanced multitrait PRS for CAD was built using MTAG method and its performance was compared to a traditional CAD PRS by testing associations with CAD in an independent sample (UKBB, *n* = 343,676).

Our work is a systematic study of genetic effects on medication-use patterns using cardiometabolic medications as a model. We test three main hypotheses: (1) medication data can be used to identify genetic factors underlying cardiometabolic diseases, (2) genome-wide data inform us about the likelihood of medication switching and stopping, and (3) medication-use-associated genetic variation allows for enhancing polygenic prediction. We study three fundamental medication-use patterns: the cumulative medication use, medication switching and treatment discontinuation. We develop polygenic risk scores (PRS) to predict medication patterns and use medication data to identify risk factors for cardiometabolic diseases. We conduct a genetic population-based biobank study comprising up to 193,933 Finnish participants of the FinnGen study^30^ linked to the nationwide drug purchase registry covering every prescription drug purchase in Finland since 1995. We perform GWAS for patterns of medication use on (1) the total number of drug purchases during the follow-up, (2) switching medications within the same therapeutic class and (3) discontinuation of the use, and fine-map our findings to a single-variant resolution. We meta-analyze our GWAS results using 184,892 participants in the Estonian Biobank (EstBB)^31^ and 188,846 participants in the UKBB^32^. We evaluate the effect of genome-wide PRS for low-density lipoprotein (LDL), systolic blood pressure (SBP) and T2D on medication use. Finally, we build a medication-use-enhanced multitrait PRS for coronary artery disease (CAD) (Fig. 1).

## Results

### Study population and medication-use patterns

FinnGen release 5 (ref. ^30^) (www.finngen.fi/en) consisted of 218,792 genotyped individuals of Finnish ancestry (Supplementary Figs. 1–4) with 5,118,565 years of drug-registry-based follow-up. In total, 56.5% were women and the mean age at the end of the follow-up was 59.8 years*.* Overall, 44,343,661 drug purchases were recorded and 3,650,495 (8.2%) of these were drugs used in treating hyperlipidemia, hypertension or T2D.

**Extended Data Fig. 1:**
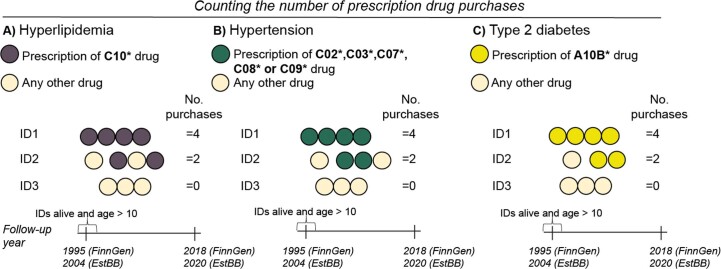
Drug use quantitative phenotypes. Drugs used in the treatment of **a**) Hyperlipidemia **b**) Hypertension and **c**) Type 2 diabetes. Dots indicate the prescriptions and text in bold indicates the drug ATC code for the specific phenotype. We added all the single purchases in underlying drug groups, the non-users were included with 0 purchases of the specific drug. Participants dead before or less than 10 years old at the start of follow-up (Jan 1st, 1995 for FinnGen and Jan 1st, 2004 for EstBB) were excluded from the analysis.

We identified all participants’ complete purchase histories of drugs targeting cardiometabolic risk factors: hyperlipidemia, hypertension and T2D. For each disorder, we used the sum of the total number of purchases of the drugs to quantify the magnitude of the pharmacological intervention (Extended Data Fig. 1) and every purchase of any package of any drug coded with an appropriate Anatomical Therapeutic Chemical Classification (ATC) code during the study period (from the start of the drug purchase registry, 1 January 1995, until death or the end of follow-up, 31 December 2018) increased the sum by one. Multiple factors, including the age of disease onset, cardiometabolic disease severity, drug resistance, perceived cardiometabolic risk and adherence, all affect this proxy of overall cumulative medication use. We included 193,933 participants (55.3% women), being at least 10 years old and alive on 1 January 1995 (Supplementary Table 1a). In total, 77,439, 121,491 and 31,223 participants recorded at least one purchase of drugs used in treating hyperlipidemia, hypertension and T2D, respectively. The longest treatment durations (mean = 11.7 years, standard deviation, s.d., = 7.97) and most purchases were seen in hypertension (mean = 69.1, excluding participants without purchases, s.d. = 67.6, median = 50). The shortest treatments were seen in T2D (mean = 8.05 years, s.d. = 6.35) and the fewest purchases in hyperlipidemia (mean = 30.7, s.d. = 27.1, median = 25) (Supplementary Table 1b).

**Extended Data Fig. 2:**
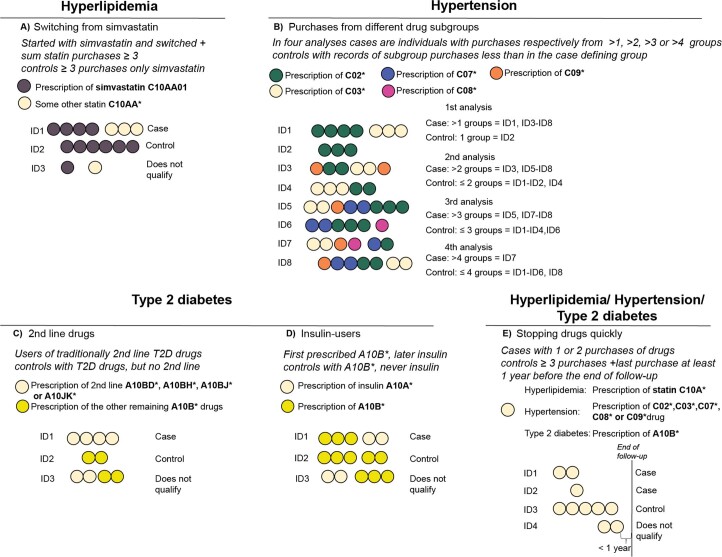
Drug use binary phenotypes. Drugs used in the treatment of **a, e**) Hyperlipidemia **b, e**) Hypertension and **c-e**) Type 2 diabetes.Dots indicate the prescriptions and text in bold indicates the drug ATC code for the specific phenotype. **a**) Cases for simvastatin switching were defined as individuals who started statin treatment with simvastatin but were later prescribed another statin and control participants who purchased only simvastatin. At least 3 purchases of statins were required for both the cases and controls. **b**) Cases for the analysis of four thresholds of drugs purchased from different hypertension medicine subgroups were defined as participants with purchases of drugs from >1, >2, >3, and >4 different hypertension drug subgroups. Controls were individuals with records of subgroup purchases that are less than in the case-defining groups. **c**) For the analysis of T2D second-line treatments, cases were defined as individuals with prescriptions of A10BD*, A10BH*, A10BJ*, or A10JK* and controls with prescriptions of any of the remaining A10B* drugs. Individuals with prescriptions for both did not qualify for the analysis. **d**) In the analysis of insulin users, cases are first prescribed A10B* (non-insulin diabetes drugs) and later A10A* (insulin) and controls only with A10B* prescriptions. Participants with first insulin prescriptions and later A10B* did not qualify. **e**) For the analysis of rapid stop of drug use done separately for the drug use phenotypes, cases were participants with only 1 or 2 prescriptions of the specific drug group and controls with 3 or more prescriptions. Individuals with the last purchase less than 1 year before the end of follow-up did not qualify for the analysis.

**Extended Data Fig. 3:**
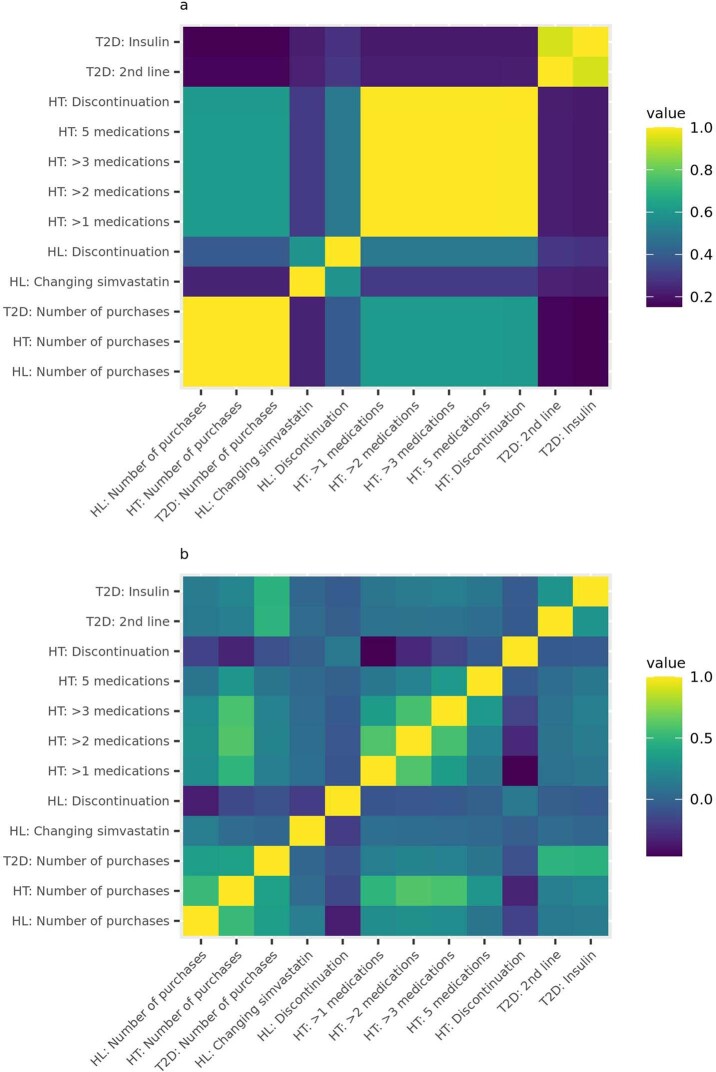
Correlation metrics between the medication use pattern phenotypes. HL = Hyperlipidemia, HT = Hypertension, T2D = Type 2 Diabetes. **a**) Sample overlap displayed by the Jaccard Index (the intersection of samples divided by the union of samples) for each phenotype. **b**) Correlation between each phenotype combination calculated in the overlapping sample intersection (Pearson’s correlation coefficient for continuous-continuous phenotype pairs, Point-biserial correlation coefficient for continuous-binary pairs, and Phi-coefficient for binary-binary pairs.

We identified common binary patterns in medication use (Extended Data Fig. 2). Although simvastatin was the most common first-choice statin, with 45,134 participants (59.0%) starting their treatment with it, 19,228 (42.6%) switched to another statin. Early discontinuation of statin use was frequent: 7,459 participants (9.86%) stopped after one or two purchases, comparable with a previous estimate^33^. In hypertension, 82,551 (65.7%) had purchases in more than one and 2,900 (2.3%) in all five ATC-based medication subgroups. In T2D, 14,292 (45.1%) recorded purchases of second-line treatments and 6,861 (22.9%) progressed to use insulin (Table 1; for EstBB and UKBB, see Supplementary Table 2). Total purchases and medication changes were positively correlated (*r* = 0.01–0.60) with one another, and each of these was negatively correlated with medication discontinuation (*r* *=* −0.46 to −0.03) (Extended Data Fig. 3).

**Table 1:** Medication patterns in hyperlipidemia, hypertension and T2D derived from the nationwide drug purchase registry in FinnGen

	ATC codes	*n*	Patients	Controls
Treatment of hyperlipidemia				
(1) Drugs targeting hyperlipidemia, number of purchases	C10	193,933		
Participants with at least one purchase		77,439		
(2) Changing simvastatin to another statin	C10AA01	45,134	19,228	25,906
(3) Fast discontinuation of statin use	C10AA	76,499	7,459	68,990
Treatment of hypertension				
(4) Drugs targeting hypertension, number of purchases	C02, C03, C07, C08, C09	193,933		
Participants with at least one purchase		121,491		
(5) More than one antihypertensive group		125,586	82,551	43,035
(6) More than two antihypertensive groups		125,586	50,482	75,104
(7) More than three antihypertensive groups		125,586	22,476	103,110
(8) All five antihypertensive groups		125,586	2,900	122,686
(9) Fast discontinuation of hypertension medication use		125,212	14,661	110,551
Treatment of T2D				
(10) Drugs targeting T2D, number of purchases	A10B	193,933		
Participants with at least one purchase		31,223		
(11) Use of second-line agents in T2D	A10BD, A10BH, A10BJ, A10BK	31,665	14,292	17,373
(12) Use of insulin	A10A	29,990	6,861	23,129

The total number of participants included in the analyses, and the number of cases and controls for the binary traits, are presented.

### Genome-wide association analyses in FinnGen

**Extended Data Fig. 4:**
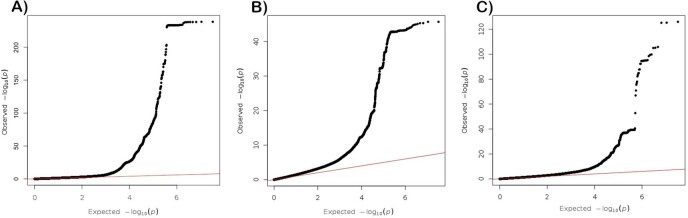
QQ-plots, number of purchases (FinnGen). Two-sided *-log*_*10*_-transformed P values of quantitative SAIGE mixed-model GWAS analyses for the total number of prescriptions of **a**) Drugs used in the treatment of hyperlipidemia **b**) Drugs used in the treatment of hypertension **c**) Drugs used in the treatment of type 2 diabetes (excluding insulins) in 193,933 FinnGen R5 participants.

**Extended Data Fig. 5:**
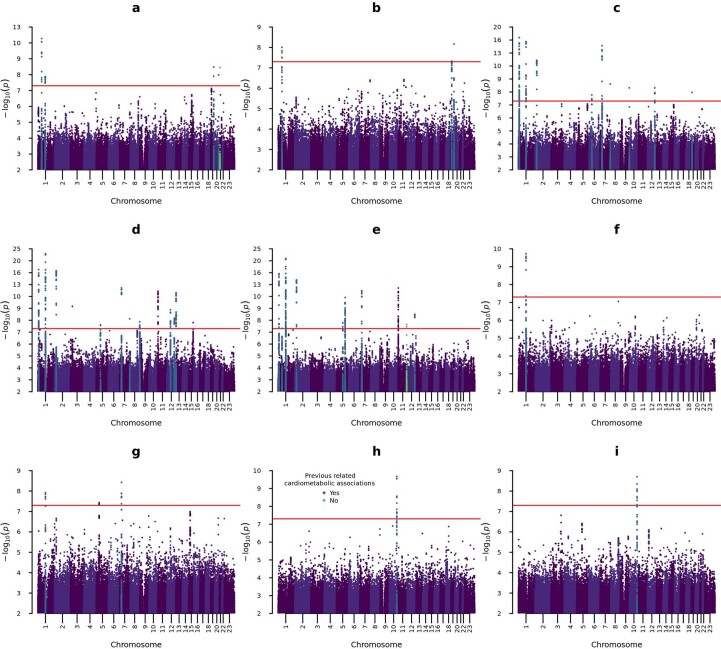
Manhattan plots, binary analyses (FinnGen). Manhattan plots, binary analyses (FinnGen). Two-sided -log10-transformed P values of binary SAIGE mixed-model GWASs for **a**) Changing simvastatin to another statin **b**) Discontinuation of statin use **c**) Hypertension medications from more than 1 different therapeutic group **d**) Hypertension medications from more than 2 different therapeutic groups **e**) Hypertension medications from more than 3 different therapeutic groups **f**) Hypertension medications from all 5 therapeutic groups **g**) Discontinuation of hypertension medication use **h**) Using 2nd line type 2 diabetes medications **i**) Using insulin in type 2 diabetes. All loci containing ≥1 95% credible set(s) are highlighted related to their previously reported related cardiometabolic associations; loci that have been previously associated with lipid-related traits (**a-b**), blood pressure-related traits (**c-g**), or blood glucose-related traits (**h-i**) in blue and potentially novel associations in green. The red horizontal line signifies genome-wide significance (p < 5 × 10-8) without multiple testing corrections.

**Extended Data Fig. 6:**
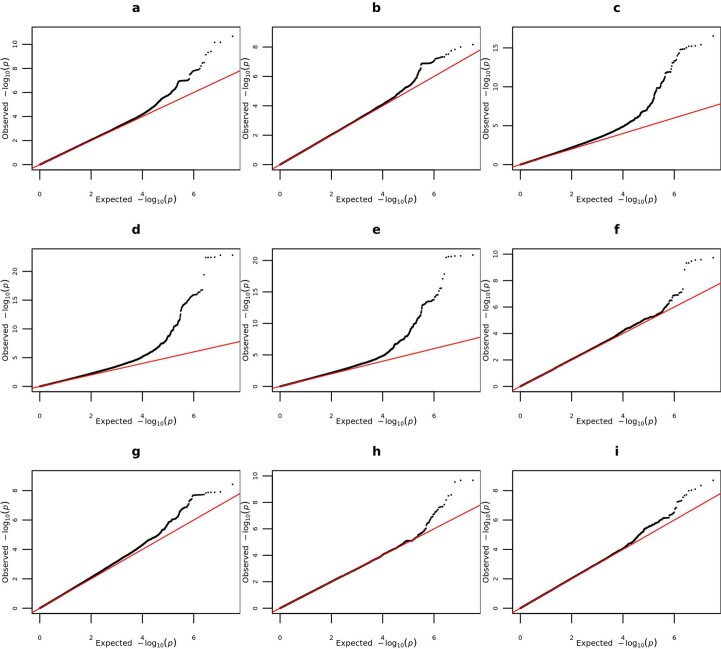
QQ-plots, binary analyses (FinnGen). Two-sided *-log*_*10*_-transformed P values of binary SAIGE mixed-model GWASs for **a**) Changing simvastatin to another statin **b**) Discontinuation of statin use **c**) Discontinuation of hypertension medication use **d**) Hypertension medications from more than 1 different therapeutic group **e**) Hypertension medications from more than 2 different therapeutic groups **f**) Hypertension medications from more than 3 different therapeutic groups **g**) Hypertension medications from all 4 therapeutic groups **h**) Using 2nd line type 2 diabetes medications **i**) Using insulin in type 2 diabetes. The error bands indicate 95% confidence intervals for the quantiles of a normal distribution.

We conducted GWAS for the 12 medication-use phenotypes (Table 1) of total purchases, medication switching and discontinuation of up to 24 years of data (mean = 23.4, s.d. = 2.68) on hyperlipidemia, hypertension and T2D medications in FinnGen. Of 16.4 × 10^6^ variants (10.3 × 10^3^ with minor allele frequency (MAF) > 0.005 included in quantitative analyses), 23,577 were genome-wide significant (GWS, *P* < 5 × 10^−8^) in at least one analysis, with 621 1 Mb windows of genome-wide significant associations to medication-use phenotypes (Manhattan plots for FinnGen GWAS: https://meds.finngen.fi). Combining all GWS variants from all analyses within 1.5 Mb windows resulted in 303 independent loci: 91 with leading associations (smallest *P* values) for hyperlipidemia, 145 for hypertension and 67 for T2D medication patterns. In total, 298 loci had leading associations for the quantitative and five for the binary medication phenotypes (Extended Data Figs. 4–6 and Supplementary Table 3).

**Extended Data Fig. 7:**
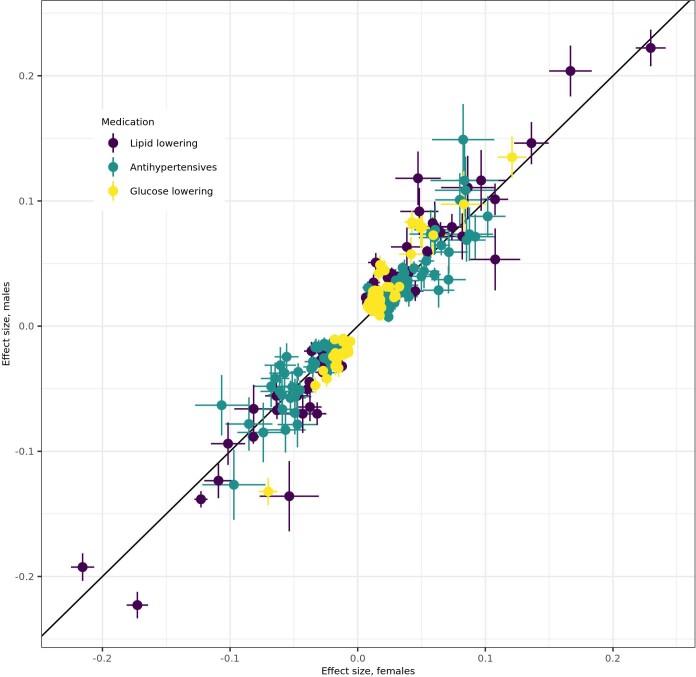
Effect sizes in sex-stratified genome-wide association analyses. Effect sizes (regression coefficient betas) with 95% confidence intervals (error bars) of the lead variants in genome-wide significant loci (in the analyses of all participants) for females (n = 107,231) and males (n = 86,702) in sex-stratified genome-wide analyses for the total number of prescriptions of drugs used in the treatment of hyperlipidemia, and type 2 diabetes (excluding insulins) in 193,933 FinnGen R5 participants.

Secondary sensitivity GWAS displayed overlapping GWS loci (case-control analyses of ever having purchased the studied drugs: 132 overlapping/2 additional GWS loci, annual purchases: 225/7, total purchases, excluding participants without any purchases: 73/5; Supplementary Tables 4–7). All linkage disequilibrium score regression (LDSC)^34,35^ genetic correlations (RGs) between the initial and the sensitivity analyses were close to 1 (Supplementary Table 8). Sex-stratified GWAS of total purchases (*n*_women_ = 107,231, *n*_men_ = 86,702) supported shared genetic architectures between the sexes as RGs between sexes were approximately 1 for all medications (hyperlipidemia, hypertension, T2D) and effect sizes for the 298 GWS lead variants were highly correlated (correlation coefficient for lead variant effect sizes (*r*_*β*_) = 0.95) between sexes (Supplementary Table 9 and Extended Data Fig. 7). Finally, we performed interaction analyses for the 303 lead variants, introducing lead variant allele (G) × age or G × follow-up time interaction terms in the models. Interactions with age were significant (*P* < 0.05/303 GWS lead variants) for 37 variants (Supplementary Table 10), whereas there were no significant interactions with follow-up time.

### Meta-analyses in FinnGen, the EstBB and the UKBB

In GWAS for all 12 medication-use patterns in EstBB with medication purchase data (*n*_*EstBB*_ = 184,892, binary phenotypes *n* = 5,067–74,699) and three medication quantity analyses using prescription data in UKBB (*n*_UKBB_ = 188,846), we saw highly concordant effect directions (251 out of 288 lead variants in FinnGen were concordant in all samples, *P* value from binomial test (*P*_binom_) = 6.0 × 10^−111^; Supplementary Table 3). We performed meta-analyses of all three samples for the number of purchases (*n*_total_ = 567,671), of FinnGen and EstBB for the binary phenotypes (*n*_total_ = 42,332–200,285) for all loci with a suspected association in FinnGen (*P* < 5 × 10^−6^). In total, 333 independent loci were associated with at least one medication phenotype (74 with leading association for hyperlipidemia, 181 for hypertension and 78 for T2D), with a stricter criterion for genome-wide significance (*P* < 5 × 10^−9^), including 94 loci not significant (*P* ≥ 5 × 10^−8^) in FinnGen (Supplementary Table 11).

### Fine-mapping in FinnGen

Fine-mapping the significant (*P* < 5 × 10^−8^) 480 locus–medication associations (a locus may be associated with multiple phenotypes) in FinnGen using the sum of single effects method^36^ resulted in 494 95% credible sets (CS) in 347 locus–medication associations. In 73 CS the variant with the highest posterior probability for causality was at least twofold Finnish-enriched compared with non-Finnish-Swedish-Estonian European populations. In total, 81 CS included one or more functional missense or predicted loss-of-function variants (Supplementary Tables 12–18). Overall, 448 CS overlapped with associations reported previously for the underlying trait (lipids, blood pressure and fasting glucose levels), whereas 40 distinct loci (with 46 CS) had no previous GWS associations in external summary statistics and the GWAS catalog.

### CS for total number of medication purchases

**Fig. 2:**
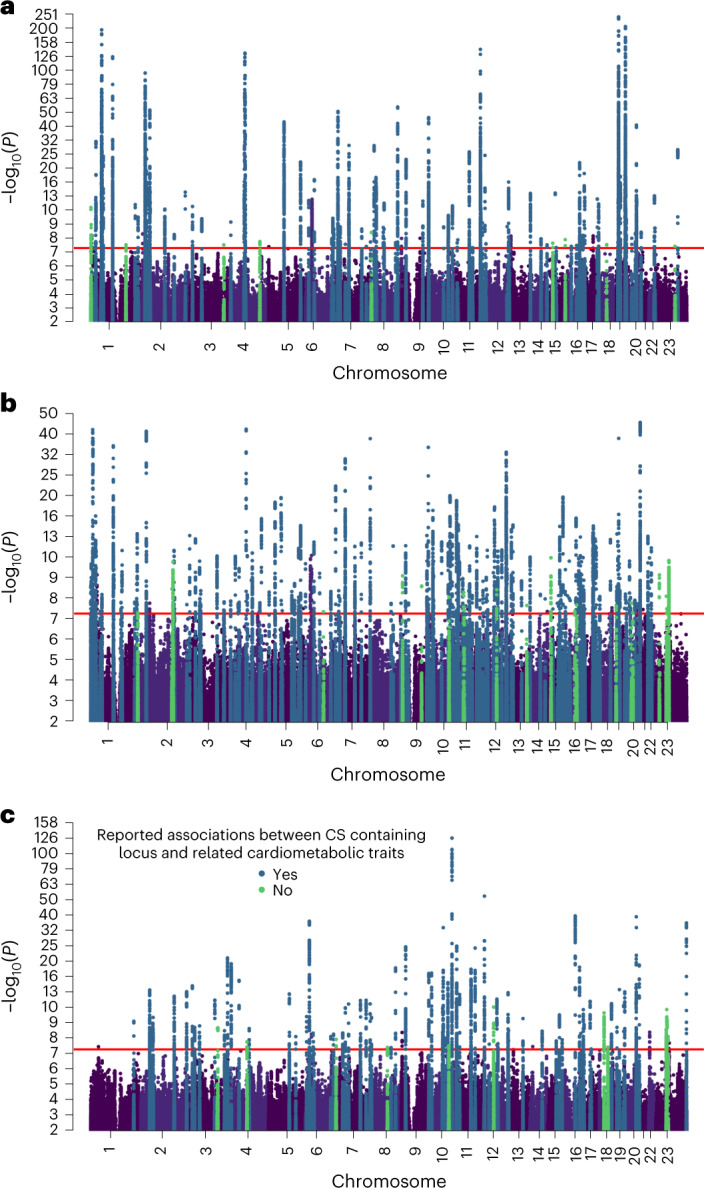
GWAS association analysis results for the total number of medication purchases in FinnGen. **a**–**c**, Manhattan plots with two-sided *P* values of quantitative SAIGE mixed-model GWAS (*n* = 193,933) for the number of recorded purchases of drugs used in the treatment of hyperlipidemia (**a**), hypertension (**b**) and T2D (**c**). All loci containing one or more 95% CS are highlighted according to their previously reported related cardiometabolic associations; loci that have been previously associated with lipid-related traits (**a**), blood pressure-related traits (**b**) or blood glucose-related traits (**c**). The horizontal line signifies genome-wide significance (*P* < 5 × 10^−8^) without additional multiple testing correction.

In total, 450 CS were associated with the overall number of purchases of drugs used in the treatment of hyperlipidemia (83 loci, 137 CS), hypertension (145 loci, 221 CS) and T2D (77 loci, 92 CS) (Fig. 2, Extended Data Fig. 4 and Supplementary Tables 14–16). Of loci, 255 were GWS in the meta-analysis of FinnGen, UKBB and EstBB (*P* < 5 × 10^−9^) (Supplementary Table 12). Most CS (85.2%) overlap with previously associated loci for cardiometabolic risk factors: variants elevating risk factor levels were associated with more purchases and risk factors that lowered variants with fewer purchases. There were 38 loci containing 44 CS (including six missense variants) that had no related previous GWS associations (Supplementary Tables 14–16).

The strongest associations (*P*_lead variant_ < 10^−100^, Bayes factor (BF)_CS_ > 10^100^) for hyperlipidemia medications were in established lipid loci (*APOE*, *LDLR*, *PCSK9*, *APOC1*, *CBLC* and *ANKRD17*). The 137 CS included 29 functional variants (28 missense, one predicted loss of function). Nine loci had no previous lipid-related associations (Supplementary Table 14).

In total, 221 CS associated with hypertension medication purchases contained 49 functional variants. There were 20 loci that had no associations with blood pressure (Supplementary Table 15). Overall, 92 CS associated with T2D medication purchases contained 21 functional variants. Of 11 loci that had not been previously associated with glucose-related traits, four contained a Finnish-enriched CS lead variant (more than threefold) (Supplementary Table 16).

### CS for changes in medication use

CS in four established lipid loci (*PCSK9*, *CELSR2*, *APOE* and *CHD4*) were associated with switching simvastatin to another statin with shared variant effect directions for LDL. A locus (*P* value in meta-analysis (*P*_meta_) = 2.6 × 10^−9^) without previous cardiometabolic associations (nearest gene: AP000472.2) contained a 6.4-fold Finnish-enriched lead variant (*21:22082072:G:A*, MAF = 8.0 × 10^−4^) (Extended Data Fig. 5a and Supplementary Tables 12 and 17).

In total, 32 CS were associated with purchasing hypertension medications from different ATC-based medication groups (Table 1). One locus (*WNT2B*) was associated with purchases from all four analyzed thresholds of the number of antihypertensive drug groups (more than one, two, three or four groups). Three loci (*HOXA13*, *CASZ1*, *KCNK3*) were associated with three thresholds (more than one, two and three groups) (Extended Data Fig. 5c–f and Supplementary Tables 12and 17).

A previously reported T2D locus (*TCF7L2*) was associated with both using second-line T2D treatments (*P*_meta_ = 2.1 × 10^−13^) and insulin (*P*_meta_ = 2.3 × 10^−11^) (one CS per analysis) (Extended Data Fig. 5h,i and Supplementary Tables 12 and17).

Out of 37 CS containing loci–phenotype associations for medication changing, 24 were significant in meta-analyses (*P* < 5 × 10^−9^) and 36 had concordant effect directions in FinnGen and EstBB (*P*_binom_ = 7.3 × 10^−12^) (Supplementary Table 12).

### CS for discontinuation of medication use

CS in known lipid- (*PCSK9*, *LDLR* and *APOE*) and blood pressure-related (*WNT2B* and *HOXA13*) loci were associated with discontinuation of medication use in hyperlipidemia (Extended Data Fig. 5b and Supplementary Table 18) and hypertension (Extended Data Fig. 5g and Supplementary Table 18), respectively. The lead variants had the opposite effect to the underlying related risk factors, number of medication purchases and medication changing.

### Medication-use-specific associations and cardiometabolic risk

We analyzed whether the 40 medication-use-associated loci (with 46 CS) without previous GWS associations for related cardiometabolic traits would indicate cardiometabolic or medication-specific links. Of these loci (20 CS), 18 were associated (*P* < 5 × 10^−9^) in the meta-analyses of FinnGen, EstBB and UKBB and the remaining 22 loci had seven CS with at least a twofold Finnish-enriched lead variant (Supplementary Table 19). We compared hyperlipidemia, hypertension and T2D medication associations with LDL^18^, SBP^19^ and T2D^21^ in previous GWAS, respectively. In total, 16 of the 35 lead variants present in external disease-related summary statistics were associated with the risk factors (multiple comparison adjusted *P* (*P*_adj_) < 0.05 at false discovery rate (FDR) 5%), whereas 29 had shared effect directions (*P*_binom_ = 7.0 × 10^−7^) (Supplementary Table 20). Eight were associated with CAD (*P*_adj_ < 0.05 at FDR 5%) in a meta-analysis of FinnGen, UKBB and CARDIoGRAMplusC4D^37^ (*n*_cases_ = 113,168, *n*_total_ = 811,555) and 35 had concordant effect directions (*P*_binom_ = 9.3 × 10^−8^) (Supplementary Table 21). Overall, 16 of the CS lead variants were associated (*P* < 0.05 out of 231 endpoints) with at least one cardiometabolic disease in a list of more detailed endpoints in FinnGen (Supplementary Table 22).

**Fig. 3:**
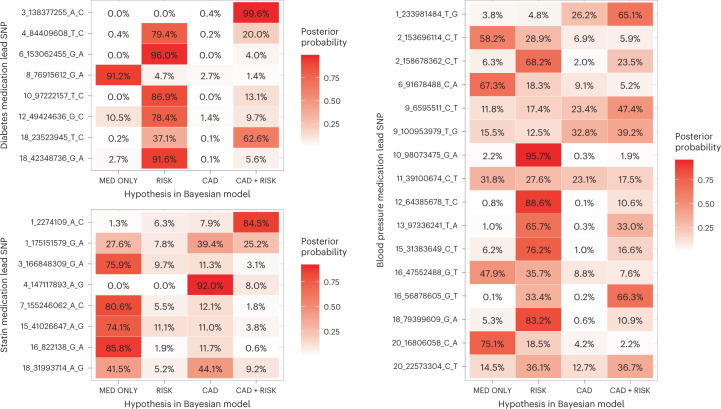
Shared effect between medically treated cardiometabolic risk factor, CAD and lead SNPs from GWAS of the total number of purchases of medications for hyperlipidemia, hypertension and T2D. Bayesian posterior probabilities of hypothetical models are displayed on the *x* axis for the number of purchases of T2D (upper left), hyperlipidemia (lower left) and hypertension (right) medications lead SNPs (*y* axis). MED ONLY, SNP affects medication use only model; RISK, correlated effect model across medication use and risk factor but not CAD; CAD, correlated effect model across medication use and CAD but not the risk factor; CAD + RISK, correlated effect model across medication use, risk factor and CAD. The variant association statistics used to compute the posterior probabilities for LDL, SBP and T2D and came from previous studies^20,21,23^ with sample sizes of 340,951, 757,601 and 898,130, respectively. For CAD, a meta-analysis including FinnGen, UKBB and CARDIoGRAMplusC4D^37^ (*n* = 811,555)^40^ was used.

Finally, we used a Bayesian framework to assess shared effect with the underlying risk factor (LDL, SBP, T2D) and CAD in the 32 novel autosomal associations for medication purchases. We found a likely CAD association (posterior inclusion probability (PIP) > 60%) in seven loci, including a locus (*chr4:147117893:A:G/TTC29*) associated with CAD and hyperlipidemia medication, but not with the underlying risk factor (LDL). Seven loci did not associate with either the considered risk factor or CAD (PIP > 60% for association with medication use only) (Fig. 3).

### Genetic correlations between medication use and cardiometabolic risk

**Fig. 4:**
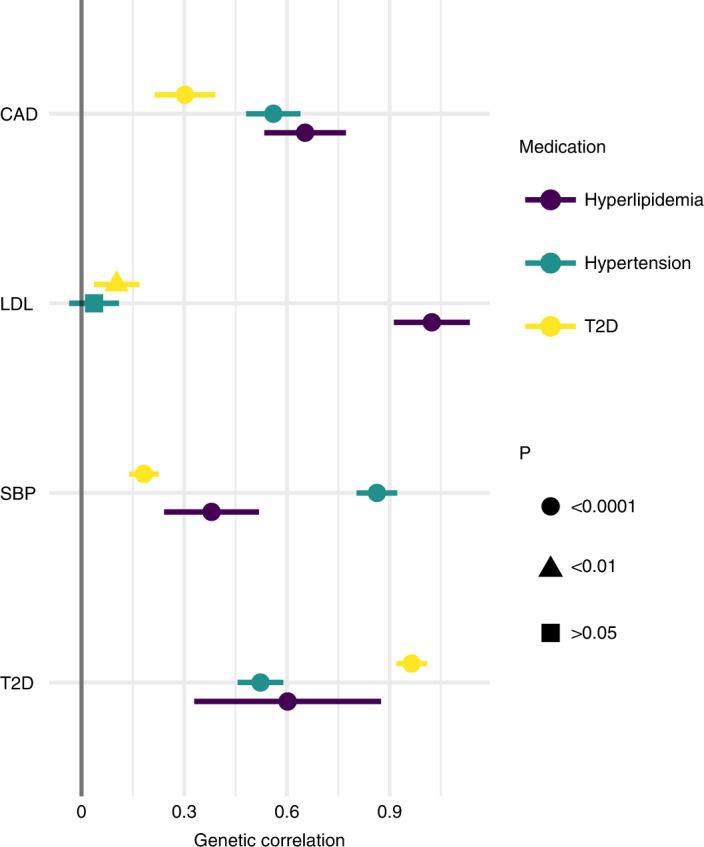
Genetic correlations between total numbers of drugs purchased for cardiometabolic indications and the underlying cardiometabolic risk factors and CAD. Genetic correlations between the total number of hyperlipidemia, hypertension and T2D medication purchases (*n* = 193,933) and CAD (CARDIoGRAMplusC4D^37^, *n* = 194,427), LDL^18^ (*n* = 340,951), SBP^19^ (*n* = 757,601) and T2D^21^ (*n* = 898,130) estimated with LDSC. The association statistics for LDL, SBP, T2D and CAD used to compute genetic correlations came from previous studies. Point estimates of genetic correlation and their 95% CI, indicated using error bars, are presented. The genetic correlation between LDL and the total number of hypertension medications was not significant, whereas all other genetic correlations were significant (two-sided *P* < 0.05 at 5% FDR).

Using LDSC^34,35^, we report (*P* < 0.05 at 0.05 FDR) genetic correlation (RG) between the medication use and the underlying cardiometabolic risk (RG approximately 0.9–1.0). Medication use was correlated with CAD (Fig. 4). This is in line with 158 of the 333 medication-use-associated loci (*P* < 5 × 10^−9^) being associated with CAD (*P*_adj_ < 0.05 at FDR < 0.05, shared direction of beta; Supplementary Table 23).

**Extended Data Fig. 8:**
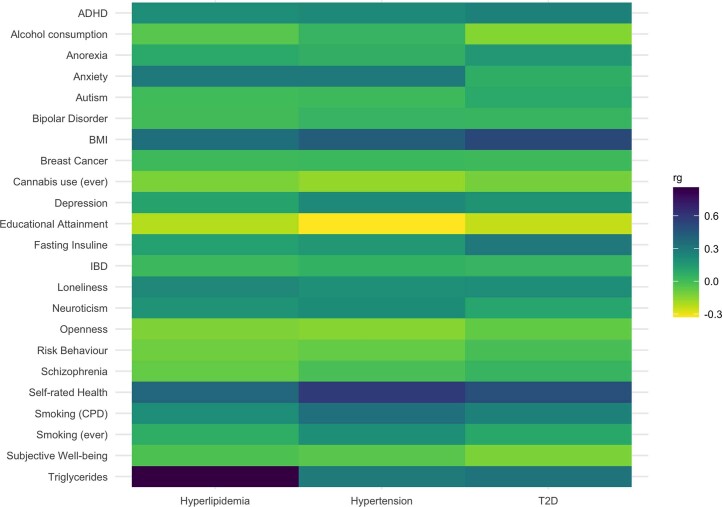
LDSC genetic correlations. Linkage disequilibrium score regression (LDSC) genetic correlation estimates: the total number of medication purchases (hyperlipidemia, hypertension, and type 2 diabetes) and selected cardiometabolic and socioeconomic, psychological, and psychiatric traits.

**Extended Data Fig. 9:**
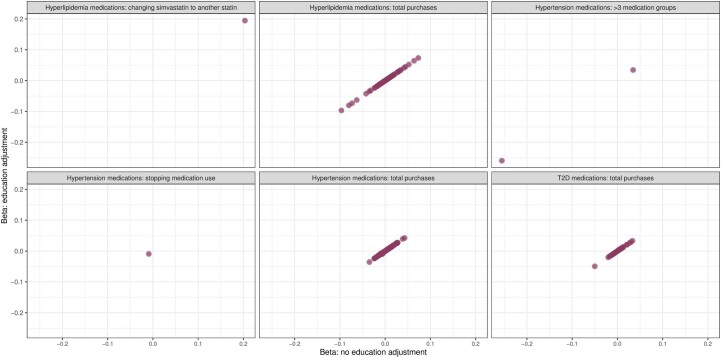
Betas in EstBB without and with educational attainment. Effects of the independent 289 medication use lead association (in FinnGen) found in EstBB without (x-axis) and with (y-axis) the inclusion of educational attainment as a covariate in the GWAS models. Including educational attainment had no effect on either effect directions or sizes.

Of the non-cardiometabolic conditions, ADHD, depression, educational attainment, loneliness, personality trait neuroticism and self-related health were all correlated with all three medication-use phenotypes (medication GWAS summary statistics were from FinnGen, others from external sources; Supplementary Table 24 and Extended Data Fig. 8). In a sensitivity GWAS in EstBB (*n* = 132,982–132,987) adjusting for educational attainment (a proxy for socioeconomic status), we did not observe any change in effect sizes or statistical significance levels of the GWS lead variants (Supplementary Table 25 and Extended Data Fig. 9).

### Polygenic cardiometabolic risk associates with medicine use

**Fig. 5:**
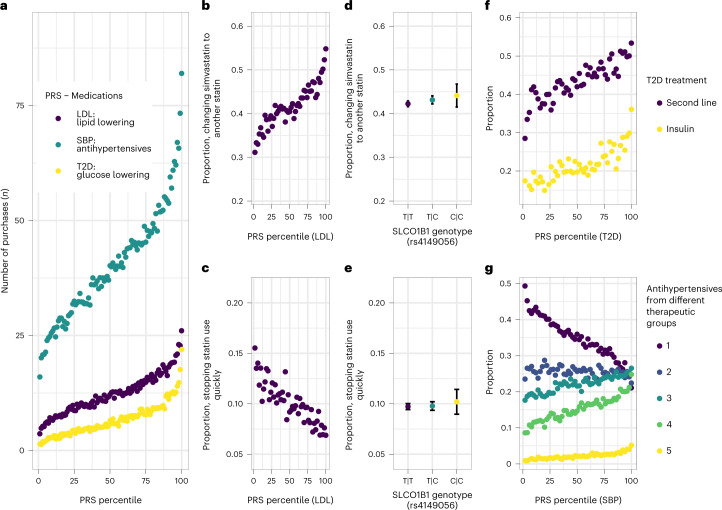
Associations between cardiovascular risk-factor PRS and medication-use patterns. Associations between PRS for LDL, SBP and T2D and medication-use patterns of hyperlipidemia, hypertension and T2D are presented, respectively. **a**, Associations between PRS percentiles and the number of medication purchases (LDL PRS and hyperlipidemia medications, SBP PRS and hypertension medications, T2D PRS and T2D medications, *n* = 193,933). **b**–**e**, LDL PRS and prevalence of changing simvastatin to another statin (*n* = 45,134) (**b**) and discontinuation of statin use (*n* = 76,499) (**c**), in comparison with differences in statin-associated myopathy-related SLCO1B1 genotypes (*n* = 45,134, *P* = 0.11 (**d**); *n* = 76,499, *P* = 0.22 (**e**)). **f**, Associations between T2D PRS and use of second-line T2D treatments (*n* = 31,665) and insulin (*n* = 29,990). **g**, Association between SBP PRS and use of hypertension medications from different numbers of distinct medication groups (*n* = 125,586). PRS are split into bins of 1% (**a)** and 2% (**b**,**c**,**f**,**g**). The LDL, SBP and T2D PRS were computed from the GWAS association statistics from previous studies^18,19,21^ with sample sizes of 340,951, 757,601 and 898,130, respectively. The error bars signify 95% CI.

The PRS of LDL, SBP and T2D were strongly associated with the related medication-use patterns (Fig. 5 and Supplementary Table 26). Participants in the highest decile recorded, on average, 15 (linear regression coefficient (beta) = 14.6; 95% confidence interval (95% CI) = 14.2–15.0), 42 (beta = 42.0; 95% CI = 40.9–43.1) and 12 (beta = 11.9; 95% CI = 11.5–12.3) more hyperlipidemia (mean number of purchases = 12.2; s.d. = 22.8), hypertension (mean = 40.1; s.d. = 61.6) and T2D purchases (mean = 6.54; s.d. = 21.9) compared with the bottom decile, respectively. In the top decile of the LDL PRS, the odds of switching simvastatin to another statin were 103% higher (odds ratio (OR) = 2.03; 95% CI = 1.85–2.23) than in the bottom decile. In contrast, the odds of discontinuing statin use were lower in the top decile (OR = 0.421; 95% CI = 0.376–0.473). For the top decile of the SBP PRS, the odds of using more than one group of antihypertensives were 210% higher (OR = 3.11; 95% CI = 2.93–3.31) than in the bottom decile and the odds of having used all five groups of antihypertensives were 320% higher (OR = 4.21; 95% CI = 3.42–5.17). In contrast, the odds of discontinuing hypertension medication were 70% lower (OR = 0.305; 95% CI = 0.280–0.333) in the top decile. Finally, individuals in the top decile of the T2D PRS had 160% higher odds of using insulin (OR = 2.62; 95% CI = 2.21–3.10) and 120% higher odds to use second-line treatments (OR = 2.23; 95% CI = 1.96–2.54).

Participants in the top deciles of the PRS started medication use 3.9–6.3 years earlier than in the bottom deciles. All associations remained significant in analyses adjusting for the age of treatment onset (Supplementary Table 26).

A missense variant rs4149056 in *SLCO1B1* has previously been associated with statin-induced myopathy^26^. We did not observe effect to statin discontinuation (OR, homozygous carriers versus non-carriers = 1.08; 95% CI = 0.957–1.220; *P* = 0.22) or switching simvastatin (OR = 1.08; 95% CI = 0.982–1.190; *P* = 0.11). There was a small additive effect for total lipid-lowering drug purchases (OR = 0.988; 95% CI = 0.982–0.995; *P* = 0.0004) and changing (OR = 1.04; 95% CI = 1.00–1.07; *P* = 0.03) but not for discontinuation (OR = 1.02; 95% CI = 0.978–1.020; *P* = 0.33). We summarize results for previously reported pharmacogenomic variants in Supplementary Table 27.

### Medication-use-enhanced polygenic risk score for CAD

We used multitrait analysis of GWAS (MTAG)^38^ to perform a joint analysis of CAD and the number of drug purchases for hyperlipidemia, hypertension and T2D, restricting analyses to HapMap 3 single-nucleotide polymorphisms (SNPs)^39^. We built two PRS (CAD PRS and MTAG-CAD PRS) from the two GWAS summary statistics. Both PRS were associated with CAD in the UKBB (17,986 cases, *n* = 343,676). Comparing the top 1% to the middle quintile, OR = 4.40 for CAD PRS (95% CI = 3.96–4.89) and 4.78 for MTAG-CAD PRS (95% CI = 4.31–5.30). The MTAG PRS had a 22% higher pseudo-*R*^2^ for CAD (*R*^*2*^_MTAG-PRS_ = 0.0424, *R*^*2*^_PRS_ = 0.0348, Davidson–MacKinnon *J-*test *P* (*P*_J-test_) = 1.0 × 10^−208^) and a 0.6 percentage points higher area under the receiver operating characteristic curve (AUC) (AUC_MTAG-PRS_ = 0.790, AUC_PRS_ = 0.784). A similar risk factor MTAG PRS combining risks for CAD, hyperlipidemia, hypertension and T2D resulted in an OR estimate between CAD PRS and medication-enhanced MTAG-CAD PRS (OR 99% versus 1% = 4.68, 95% CI = 4.22–5.19, AUC = 0.788, *R*^*2*^ = 0.040) (Extended Data Table 1). Assessing effect of rare variants with MAF < 1% excluded by MTAG, a CAD PRS together with a traditional weighted-sum PRS based on effect sizes of the lead SNPs of the medication-use GWS loci performed equally, whether the rare variants were included (*R*^*2*^ = 0.0378) or not (*R*^*2*^ = 0.0374).

## Discussion

In this study, we demonstrate the highly polygenic nature of lifelong medication use in cardiometabolic conditions. We discovered hundreds of genetic predictors for temporal medication-use patterns: (1) the total quantity of lifelong purchases, (2) changing medication and (3) discontinuation. We report 333 independent loci associated with medication use and 495 CS with ≥95% posterior probability for causality. Whereas most associations have been previously linked to lipid, blood pressure or glucose-related traits, we discovered loci without previous cardiometabolic link. We developed polygenic predictors for medication use and showed how genetic association results for medication use can refine polygenic prediction.

Most genetic factors driving the differences in medication use in cardiometabolic conditions are shared with the underlying risk factors. Most loci associated with total purchases and medication changes (hyperlipidemia: *APOE*, *PCSK9* and *LDLR*; hypertension: *WNT3B*, *HOXA13*, *CASZ1* and *KCNK3*; T2D: *TCF7L2*) have been associated with cardiometabolic risk factors with shared effect directions^16–22^. In contrast, the associations for discontinuation (statins: *PCSK9*, *LDLR* and *APOE*; antihypertensives: *WNT3B*, *HOXA13*, *CASZ1* and *KCNK3*) had opposite effect directions. The PRS for LDL, SBP and T2D were strongly associated with medication-use patterns, supported by a reported association between T2D PRS and T2D patients’ progression to use insulin^40^. Genetic correlations were highest between total purchases and the treatment targets. In contrast, known pharmacogenetic variants associated with medication-related adverse events^26–28^, such as *SLCO1B1* increasing statin-related myopathy, were not among the leading associations. Together, these results demonstrate the central role of the underlying genetic liability in medication use.

There are several explanations for these findings. First, the total number of purchases is an aggregate phenotype combining both the length of the treatment and the number of different medications used within a certain time window. It is driven by the underlying polygenic risk for the risk factor itself associated with the age of onset^24,41^, levels of the measured risk factor^18,42,43^ and hard cardiovascular events^18,22,41,42^. Second, these factors are all driving earlier initiation of the medication, a greater number of different therapeutic agents used and the total number of medications purchased. Third, the polygenic risk for the underlying risk factor was inversely associated with treatment discontinuation.

We propose reasons for the negative association between cardiometabolic risk and treatment discontinuation. Patients with higher genetic cardiometabolic risk are at higher risk for severe cardiometabolic diseases, cardiovascular complications and a family history of CVD, all motivating the treatment, even if side effects are experienced. Medical professionals might try to find tolerated medication combinations if patients wanted to abandon the medications or side effects are experienced. Also, nonpharmacological lifestyle-related interventions are more likely sufficient^5,6^ if the genetic risk is low as higher genetic risk correlates with higher measures of cardiovascular risk factors^18,42^. Finally, we know that higher genetic risk for individual risk factors is associated with hard CVD outcomes^18,22,41,42^. In secondary prevention of CVD, the treatment is more aggressive and the stricter treatment goals are unlikely to be met without ongoing pharmacotherapy. Individuals with high inherited risk have a harder time meeting the goals and need continued treatment, whereas individuals with lower genetic risk meet the goals more easily, sometimes leading to treatment discontinuation.

In total, 40 associated loci were not previously linked with the underlying risk factors. The link between medication use and cardiovascular risk, partially independent of the risk factors, may explain why these loci had not been reported in well-powered risk factor studies^18,19,21^, because high CVD risk indicates pharmacological treatment of CVD risk, despite normal risk factor measures^5,6,44^. For example, lipid-lowering treatment is initiated earlier and targets are stricter in individuals with CVD or diabetes^6^. Whether these associations indicate medication-specific pathways for action, including drug efficacy and side effects or are more behavioral by nature, warrants further study.

A medication-enhanced multitrait PRS for CAD showed better risk discrimination compared with a traditional single-trait CAD PRS, although the improvement was not clinically meaningful. This is in line with results for combining risk factor GWAS results with CAD for polygenic prediction^45^. Rare variants may provide further predictive power to the PRS.

The study has strengths and limitations. FinnGen consisted of individuals linked to the Finnish drug purchase registry covering all drug purchases since 1995, being superior to our other samples. The Finnish bottleneck population provides for genetic discovery^46^ and 73 CS had a Finnish-enriched lead variant. Replication in datasets with enough carriers of Finnish-specific variants is therefore challenging. By combining drugs with varying mechanisms for action to maximize statistical power, the possible molecule-specific pharmacogenomic aspects might be overlooked. The medication dosage could have an effect, but dosage information was unavailable. For example, we may underestimate the effect of *SLCO1B1 rs414956* polymorphisms on medication use as the related statin-induced myopathy is dependent on simvastatin doses of at least 40 mg daily^47^. However, our quantitative phenotypes grouped together different medications and our binary trajectory design was dose independent, and we show the robust association between the underlying cardiovascular risk and the medication patterns even without adjustment for dosage. In addition, because of widespread off-label use and additional indications for many of the studied medications^48^, not all purchases were to treat cardiometabolic conditions, but our key findings display the association between the cardiometabolic risk and the studied medication patterns.

The long follow-up time in our study highlights time-dependent progression in medication-use practices, seen in the overrepresentation of simvastatin, the first widely used statin^49^, as the first-choice statin in FinnGen. However, the wide use of simvastatin allowed us to test associations on switching to other statins. Although the medication registry covers the whole nation of Finland, the biobank ascertainment may cause biases, including survival bias, because participants need to be alive until they are recruited (Supplementary Fig. 5). Given the replicability of associations, it is unlikely that these biases largely affect our main findings. Also, due to possible spectrum bias, the results from our quantile-based PRS analyses should be generalized to other populations with caution^50^. In addition, given the self-reporting of psychological and socioeconomic factors in our study, the effect of these traits may be underestimated. Finally, although the study was conducted in three independent biobanks, all our samples were of European ancestry, limiting the generalizability of our results^51,52^.

In summary, we demonstrate the highly polygenic genetic architecture of lifelong medication-use patterns in hyperlipidemia, hypertension and T2D largely shared with cardiometabolic traits. Our findings highlight the possible utility of using risk-factor-related genetic information for optimizing pharmacological treatment with medicine-use-related genetic information to improve the prediction and prevention of cardiometabolic diseases.

## Methods

### Study sample

The data comprised 218,792 Finnish individuals from FinnGen Data Freeze 5, which includes prospective epidemiological and disease-based cohorts and hospital biobank samples (Supplementary Table 28). The data were linked by the unique national personal identification numbers to national hospital discharge (available from 1968), death (from 1969), cancer (from 1953) and drug purchase (from 1995) registries. The registry data were available until 31 December 2018.

### Drug purchase registry

The Finnish drug purchase registry managed by The Social Insurance Institution of Finland (Kela) contains all prescription drug purchases starting from 1 January 1995. All prescription drug purchases are dated and coded with the national version of the World Health Organization ATC, thus enabling molecular and higher drug-group-level classifications.

### Medication-use phenotypes

We identified the analyzed drug-use phenotypes using the drug purchase registry and ATC codes (Table 1). First, we identified all purchases of drugs used in the management of three common risk factors of CVD, hyperlipidemias, hypertension and T2D, lipid-modifying agents (ATC code starts with C10), agents used in the treatment of hypertension (five different ATC groups: C02*, C03*, C07*, C08* and C09*) and blood glucose-lowering drugs, excluding insulin (ATC = A10B*).

For the analysis of quantitative phenotypes of drugs used in the treatment of hyperlipidemia, hypertension and T2D, we counted all single drug purchases in these categories for all the study participants. All the nonusers were included in these phenotypes with zero purchases (Supplementary Table 1a). To exclude age-related medicine-free follow-up from the analyses, we excluded all the participants who were dead or younger than 10 years old at the beginning of the follow-up (1 January 1995), resulting in the final sample sizes of 193,933 participants.

For the analysis of binary phenotypes, we identified risk-factor-specific common drug-use patterns from the purchase registries for hyperlipidemias, hypertension and T2D from the whole dataset of 218,588 individuals alive at the beginning of the follow-up.

Among the hyperlipidemia drug users, we identified as patients (1) those who started with simvastatin (ATC: C10AA01) but then changed to another statin (ATC: C10AA*, not C10AA01) and (2) those who started using statins but stopped the use quickly (only one to two purchases of statins, ATC = C10AA*, last purchase more than 1 year before the end of the follow-up, excluding those discontinuing later from the analysis) (Supplementary Fig. 6).

For medications used in the treatment of hypertension, we analyzed four thresholds of the number of drugs purchased from different hypertension medicine subgroups, requiring at least one purchase during the follow-up: (3) more than one group, (4) more than two groups, (5) more than three groups and (6) all five groups used. Controls were individuals with records of subgroup purchases that are less than in the case-defining groups (for example, controls for the first analysis were individuals with purchases of drugs from one hypertension drug subgroup, for the second analysis individuals with purchases from one or two hypertension drug subgroups, and so on). Discontinuation was defined by having only one or two purchases of hypertension medication, with at least 1 year of purchase free follow-up after the last purchase (Supplementary Fig. 6).

For T2D, we identified users of (7) second-line treatments (combinations: ATC = A10BD*, DPP4: ATC = A10BH*, GLP-1: ATC = A10BJ* and SGLT2: ATC = A10JK*) and (8) those starting insulin (ATC = A10A*) after initially starting with non-insulin T2D drugs.

For these drug-use patterns, only non-case users of hyperlipidemia (1 and 2), hypertension (3–6) and T2D (7 and 8) drugs were considered as controls, and those participants who never purchased the related drugs were excluded.

### Genotyping and imputation

FinnGen samples were genotyped with Illumina (Illumina Inc.) and Affymetrix arrays (Thermo Fisher Scientific). For imputation, a population-specific SISu v.3 imputation reference panel (Pärn et al., manuscript in preparation) comprising 3,775 high-coverage (25–30x) whole genomes was used (public protocol: https://www.protocols.io/view/genotype-imputation-workflow-v3-0-xbgfijw). Postimputation quality control involved nonreference concordance analyses, checking expected conformity of the imputation information (INFO)-values distribution, MAF differences between the target dataset and the imputation reference panel, and checking chromosomal continuity of the imputed genotype calls. After these steps, variants with imputation INFO score < 0.6 or MAF < 0.0001 were excluded. For details about the genotype calling, quality controls and imputation in FinnGen, see Kurki^30^.

Genotyping of DNA samples from the EstBB^31^ was done at the Core Genotyping Lab of the Institute of Genomics, University of Tartu using the Illumina Global Screening Arrays (GSA v.1.0, GSA v.2.0 and GSA v.2.0_EST). Altogether 206,448 samples were genotyped and PLINK format files were created using Illumina GenomeStudio v.2.0.4. During the quality control all individuals with call-rate < 95% or mismatching sex, which was defined based on the heterozygosity of X chromosome and sex in the phenotype data, were excluded from the analysis. Variants were filtered by call-rate over 95% and Hardy–Weinberg equilibrium *P* value < 1 × 10^−4^ (autosomal variants only). Variant positions were updated to GenomeReference Consortium Human Build 37 and all variants were changed to be from TOP strand using reference information provided by Dr. W. Rayner from the University of Oxford (https://www.well.ox.ac.uk/~wrayner/strand/). After quality control the dataset contained 202,910 samples. Before imputation, variants with MAF < 1% and indels were removed. Prephasing was done using the Eagle v.2.3 software 1 (number of conditioning haplotypes Eagle2 uses when phasing each sample was set to: –Kpbwt = 20,000) and imputation was carried out using Beagle v.28Sep18.793 (refs. ^53,54^) with an effective population size *n* = 20,000. As a reference, an Estonian population-specific imputation reference of 2,297 whole-genome sequencing samples was used^55^.

The genotyping in the UKBB was performed using the Applied Biosystems UK BiLEVE Axiom Array or the Applied Biosystems UKB Axiom Array. Imputation was done using combined Haplotype Reference Consortium, UK10K and 1000 Genomes Project phase 3 reference panels with IMPUTE4 software. For quality control, variants with INFO score ≤ 0.8, MAF ≤ 0.01 and Hardy–Weinberg equilibrium *P* value ≤ 1 × 10^−10^ were excluded. For a detailed description on genotyping, imputation and quality control in the UKBB, see Bycroft^32^.

### GWAS

For GWAS analyses, we used scalable and accurate implementation of generalized mixed model (SAIGE), which controls for unbalanced case-control ratios of binary phenotypes and sample relatedness by a two-step approach: (1) fitting the null model with the covariates age, sex, the first ten principal components and genotyping batch (for batches with at least ten cases and controls) as fixed effects and assuming the random effects are distributed as *N*(0, *τ ψ*), where *ψ* is the genetic relationship matrix and *τ* is the additive genetic variance, and (2) testing for association between each genetic variant and phenotypes by applying saddle-point approximation to the score test statistics, which obtains more accurate *P* values than the normal approximation in the presence of the unbalanced case-control ratios of binary phenotypes^56^.

For the analysis of quantitative phenotypes, we used SAIGE mixed-model linear regression. Before the final association analyses, we transformed the count data to continuous phenotypes (Supplementary Fig. 7) with the rank-based inverse normal transformation (INT) using the ‘rankNorm’ function of the R-package rNRNOmni^57^, as required (https://saigegit.github.io//SAIGE-doc/). Age, age^2^, follow-up time (end of the follow-up—1 January 1995), sex (imputed with PLINK, not included in the sex-stratified analyses), the first ten first principal components of ancestry and genotyping batch (for batches with at least ten cases and controls) were used as fixed-effect covariates. To account for the possible type I error of rare variants using INT-normalized phenotypes, we excluded variants with MAF < 0.005. Also, we excluded participants dead or under 10 years old at the beginning of the follow-up.

### Genome-wide association analyses in EstBB and UKBB

We identified the drug-use phenotypes at the EstBB in the same way as described above for the FinnGen cohort by using the drug purchasing data of biobank participants and purchased drug ATC codes. Sample sizes for genome-wide analyses were as follows. Quantitative drug purchases phenotypes: hyperlipidemia *n* = 184,880 (*n*_purchases>0_ = 28,659), hypertension *n* = 184,892 (*n*_purchases>0_ = 74,534) and T2D *n* = 184,880 (*n*_purchases>0_ = 12,795) (there were 12 new participants in the hypertension analysis because their inclusion in the EstBB was confirmed by a recorded hypertension prescription purchase); binary phenotypes switching from simvastatin: 3,013 patients and 2,054 controls; discontinuation of statins: 5,005 patients and 21,679 controls; the four thresholds of the number of drugs purchased from different hypertension medicine subgroups: (1) more than one group, 45,365 patients and 29,334 controls, (2) more than two groups, 26,490 patients and 48,209 controls, (3) more than three groups, 12,542 patients and 62,157 controls, (4) more than four groups, 3,103 patients and 71,596 controls; users of traditionally second-line T2D drugs: 3,501 patients and 9,268 controls; starting insulin after other T2D drugs: 1,728 patients and 10,614 controls.

We conducted GWAS using the SAIGE software. For the analysis of quantitative phenotypes we normalized the phenotypes with the rank-based INT using the ‘rankNorm’ function of the R-package RNOmni and used SAIGE mixed-model linear regression for genome-wide analysis, adjusting for covariates age at the end of follow-up, age at the end of follow-up squared, follow-up time (end of the follow-up to 1 January 2004), sex and the first ten principal components of the genotype matrix. All individuals who were less than 10 years old at the beginning of follow-up were excluded from the analysis. For the analysis of binary phenotypes we used SAIGE mixed-model logistic regression and adjusted for the covariates age at the end of follow-up, sex and ten principal components. For the first steps of SAIGE models we used only the genotyped data and set the parameters trace CV cutoff to 0.002, ratio CV cutoff to 0.001 and LOCO to TRUE. For the second steps of SAIGE models, we used imputed data and the parameters minMAF was set to 0.0001, minMAC to 1 and LOCO to TRUE.

In the UKBB we identified proxy drug-use phenotypes for the total number of medication purchases in the UKBB general practitioner prescription data (data field 42039) by counting all prescriptions for hyperlipidemia (British National Formulary code (BNF) = 02120*), hypertension (BNF = 0202*, 0204*, 0205* and 020602*) and T2D (BNF = 060102*) and normalizing the phenotypes. We did not consider the binary phenotypes in the UKBB because the data were restricted to prescriptions by general practitioners and there are no data available for whether the prescriptions were dispensed. The final sample was restricted to 188,846 participants, with at least one prescription of any kind in the records. We conducted GWAS using the SAIGE software. In the UKBB, instead of the lacking follow-up time covariate, we adjusted for birth year.

### Meta-analyses

GWAS analyses for (1) the total number of purchases in hyperlipidemia, hypertension and T2D in FinnGen, EstBB and UKBB, (2) binary medication purchase phenotypes in FinnGen and EstBB and (3) CAD summary statistics from FinnGen, UKBB^56^ (https://pheweb.org/UKB-SAIGE/pheno/411) and CARDIOGRAM4+^37^ were combined using an inverse-variance weighted fixed-effect meta-analysis with GWAMA v.2.2.2 (ref. ^58^). For the medication-use pattern meta-analyses, all loci (±1.5 Mb) with a potential association (*P* < 5 × 10^−6^ for the lead variant) in FinnGen were included. A stricter criterion for genome-wide significance was used (*P* < 5 × 10^−9^)^16^. For chromosomes 1–22, we included all variants that were present in FinnGen and at least one additional cohort (EstBB, UKBB or both). Chromosome X variants were only analyzed in FinnGen and interpreted genome-wide significance only if meeting the stricter meta-analysis threshold of significance (*P* < 5 × 10^−9^).

### Bayesian fine-mapping

Using GWAS summary statistics from the medication pattern analyses, we fine-mapped all regions where the lead variant reached the standard genome-wide significant *P* value of <5 × 10^−8^ in FinnGen using the sum of single effects method^36^ to allow discovery of associations driven by Finnish-enriched variants, possibly missed in other populations. Fine-mapping regions were formed using a 3 Mb window (±1.5 Mb) around each lead variant and combining overlapping regions into one. Linkage disequilibrium (LD) between each variant was calculated from individual-level FinnGen data and a minimum pairwise correlation value of 0.5 (*r*^2^ = 0.25) for variants in a CS was required. We report all 95% CS and for CS with over ten variants, we report those variants with a ≥0.01 probability of being causal. We report which CS are in loci with a genome-wide significant association for the given medication-use phenotype in the meta-analysis (*P* < 5 × 10^−9^) and which CS lead variants are Finnish enriched, with a minor allele over twofold enriched in Finns as compared to non-Finnish-Swedish-Estonian European in gnomAD v.2.0.1^59^.

### Automatized novelty reporting

To evaluate the possible novelty of the found CS associations, we compared our results to those in the GWAS catalog^60^ and the largest available GWAS summary statistics with non-overlapping samples (lipid-lowering agent patterns against statin-use adjusted LDL^18^, blood pressure-related medication patterns against hypertension medication adjusted SBP summary statistics^19^, and glucose-lowering medication patterns against T2D^21^), using a 3 Mb window (±1.5 Mb) around each 95% CS top variant. For loci with no corresponding genome-wide significant (*P* < 5 × 10^−8^) variants in the respective GWAS summary statistics, we checked all the previous associations in the GWAS catalog. If no associations related to the indication of the drugs used (lipid-modifying agents: lipid levels, lipid-level measurements and morbidities, and use of lipid-modifying agents; glucose-lowering agents: glucose-related measurements, morbidities and use of glucose altering agents; blood pressure medications: blood pressure measurements and morbidities, and use of blood pressure modifying agents) were found, we considered the association to be of potential novelty. We also report whether these possibly novel loci have been previously associated with any CVDs or any other known CVD risk factors. (For a more detailed description of the automated novelty reporting method, see https://aaltodoc.aalto.fi/handle/123456789/41629.)

### Cardiometabolic phenome-wide association study in FinnGen

For the lead variants of the CS in the possibly novel loci, we performed a cardiometabolic phenome-wide association study in FinnGen, testing the lead variants associations among 231 cardiometabolic endpoints: T1D, T2D, all endpoints from the ICD-10 main category IX ‘Diseases of the circulatory system’ with a name starting with I9_* and hyperlipidemia endpoints (E4_HYPERCHOL, E4_HYPERGLYCER, E4_HYPERLIPMIX, E4_HYPERCHYLO, E4_HYPERLIPNAS, E4_LIPODEF, E4_LIPOPROTNAS). A *P* value of <0.05/231 (the number of the considered cardiometabolic endpoints) (= 2.16 × 10^−4^) was considered statistically significant. The full list of FinnGen endpoints with detailed descriptions of their definitions (https://www.finngen.fi/en/researchers/clinical endpoints) and thorough documentation (https://finngen.gitbook.io/documentation/methods/endpoints) are available online. The publicly available results for FinnGen R5 phenome-wide association study are available online (https://r5.finngen.fi/).

### LD score regression

To estimate the genetic correlation between the medication phenotypes and the underlying risk factors we used LDSC software^34,35^. LDSC uses the LD score regression method, which quantifies the contribution of each variant by examining the relationship between test statistics and LD. We used LD scores readily calculated from the 1000 Genomes Project (EUR)^61^. To restrict to a set of common, well-imputed variants, we retained only those SNPs in the HapMap 3 reference panel^39^. The medication-use summary statistics from GWAS conducted in FinnGen were compared with summary statistics from external sources (Supplementary Table 24).

### PRS

We calculated genome-wide PRS for LDL, SBP and T2D using the PRS-continuous shrinkage priors (CS) method, a Bayesian method to infer posterior effect sizes for variants using summary statistics from GWAS and an external LD reference panel^62^. For the input weights, we used available summary statistics from external GWAS for LDL^18^, SBP^19^, T2D^21^ and a selected set of other health-related traits (Supplementary Table 28) and limited our variants to those in the HapMap 3 (ref. ^39^) reference panel. The 1000 Genomes Project (EUR)^61^ was used as an LD reference panel. Traditional PRS for medication use were calculated as effect-size weighted sums of the lead variants in the GWS loci in FinnGen (Supplementary Table 3), after pruning the summary statistics to variants in the UKBB. Two traditional weighted-sum PRS were calculated, one excluding and the other including rare variants (MAF < 1%).

### Bayesian meta-analysis

We analyzed the hypothesized shared effect of specific variants using a previously reported method of Bayesian meta-analysis^63^. This approach uses GWAS summary statistics to infer an approximate BF (ABF) for different assumptions about heterogeneity between studies, and the probability of a model is derived from its ABF relative to the sum of all model ABFs for a particular variant. We used this Bayesian framework to compare the effect between medication phenotypes in FinnGen and the respective cardiometabolic risk factors (RISK) in studies in non-Finns when the overlap between cases was negligible^18,19,21^. For CAD effect, we used the GWAS summary statistics from the meta-analysis (CAD). The models used in all tests were the null model, where the effect on RISK and CAD is zero (MED ONLY); endpoint-specific models, where the effect is assumed to be non-zero for only one endpoint (RISK or CAD); and the shared effect model, where the effect is fixed or highly correlated between RISK and CAD (CAD + RISK). In model comparison, a model was considered supported if the posterior probability of the model was ≥60%. Prior probability distribution was uniform. Analyses were performed for loci found in all summary statistics (medication use, risk factor and CAD).

### Traditional and MTAG-PRS for CAD in the UKBB

For the medication-enhanced multitrait PRS of CAD, we jointly analyzed the summary statistics for the total number of purchased medications in the treatment of hyperlipidemia, hypertension and T2D (in FinnGen) and CAD^37^ using MTAG^38^ with variants found in HapMap 3 (ref. ^39^) and all the summary statistics with a MAF of >0.01, resulting in 1,168,733 variants. The PRS were calculated using PRS-CS as described above, the traditional PRS using the CAD summary statistics and the MTAG-PRS using the MTAG-CAD summary statistics, both with the same 1,168,733 variants. For the risk factor enhanced multitrait PRS, we used a similar approach with FinnGen R5 GWAS summary statistics for hypertension (https://r5.finngen.fi/pheno/I9_HYPTENS), T2D (https://r5.finngen.fi/pheno/T2D) and a custom hyperlipidemia GWAS (cases: cases of FinnGen R5 endpoints E4_HYPERCHOL, E4_HYPERGLYCER, E4_HYPERLIPMIX, E4_HYPERLIPNAS or International Classifcation of Diseases 10th revision (ICD-10) diagnosis code E78.0–5 in the Register of Primary Health Care Visits/‘Avohilmo’, *n*_cases_ = 29,837, *n*_controls_ = 188,955).

### CAD definition in the UKBB

In the analyses testing the associations between the CAD PRS and CAD in the UKBB, CAD was defined as (1) any of I20–I25, I46 or R96 (ICD-10) as the primary or secondary cause of death (from data fields 40,001 and 40,002, age from data field 40,007), (2) any of I20.0, I21–I22 (ICD-10) or 410, 4110 (ICD-9) in the hospital inpatient records (from data fields 41,270 and 41,271), or (3) any coronary revascularization procedure (OPCS-4, variable 41,272, codes K40, K41, K42, K43, K44, K45, K46, K49, K501 and K75, and age defined based on data field 41,282; OPSC-3, data field 41,273, code 3,043, self-reported operations, data field 20,004, codes 1,070 and 1,095, with age being defined based on data field 20,010). Diagnoses dating both before and after the study recruitment were considered. The analyses were restricted to participants of White British ethnicity (self-reported, data field 21,000).

### Statistical models

R (v.3.6.0, 4.0.3, 4.1.2 and 4.1.3) was used for the analyses of age or time dependencies of the significant associations. We performed interaction analyses for the FinnGen GWS loci lead variants by introducing G × age or G × follow-up time interaction terms in the (linear or logistic) regression models, where age and follow-up time in the interaction terms were dummy variables split to be 0 or 1 by their sample medians. In addition, the same covariates were used as in the original GWAS analyses (including continuous age and follow-up time). The analyses were restricted to 134,695 non-related independent FinnGen R5 samples. For the PRS calculation, we used PLINK 2.0. R (v.4.0.0–4.1.3) was used in the analysis of PRS and drug-use patterns. Separate models were created for continuous normalized PRS and decile stratified PRS. In the analysis of associations between the PRS and the medication-use patterns, linear regression was used for the continuous traits and logistic regression for the binary traits. Age, sex, follow-up time, the first ten principal components and genotyping batch were included as covariates, and the sensitivity analyses were restricted to medication users only and adjusted for age of onset for the medication use. For the analyses studying the relationship between the PRS and the age of onset we used linear regression and included, age, sex, the first ten principal components and genotyping batch as covariates. In the analyses in the UKBB testing associations between PRS and CAD diagnosis, we used a logistic regression model with age, sex and the first ten principal components as covariates. Davidson–MacKinnon *J*-test was used to test the specification of the non-nested models^64^ and the Nagelkerke pseudo-*R*^*2*^ was used as a measure of model fit^65^. AUCs were calculated by splitting the dataset by random sampling into training (75%) and validation datasets (25%), repeated for a total of 100 rounds.

### Ethics

Patients and control subjects in FinnGen provided informed consent for biobank research, based on the Finnish Biobank Act. Alternatively, older research cohorts, collected before the start of FinnGen (in August 2017), were based on study-specific consents and later transferred to the Finnish Biobanks after approval by Valvira, the National Supervisory Authority for Welfare and Health. Recruitment protocols followed the Biobank protocols approved by Valvira. The Coordinating Ethics Committee of the Hospital District of Helsinki and Uusimaa approved the FinnGen study protocol HUS/990/2017. The FinnGen study is approved by the Finnish Institute for Health and Welfare, approval number THL/2031/6.02.00/2017, amendments THL/1101/5.05.00/2017, THL/341/6.02.00/2018, THL/2222/6.02.00/2018, THL/283/6.02.00/2019, THL/1721/5.05.00/2019, digital and population data service agency VRK43431/2017-3, VRK/6909/2018-3, VRK/4415/2019-3, the Social Insurance Institution (KELA) KELA 58/522/2017, KELA 131/522/2018, KELA 70/522/2019, KELA 98/522/2019, and Statistics Finland TK-53-1041-17. The Biobank Access Decisions for FinnGen samples and data utilized in FinnGen Data Freeze 5 include: THL Biobank BB2017_55, BB2017_111, BB2018_19, BB_2018_34, BB_2018_67, BB2018_71, BB2019_7, BB2019_8, BB2019_26, Finnish Red Cross Blood Service Biobank 7.12.2017, Helsinki Biobank HUS/359/2017, Auria Biobank AB17-5154, Biobank Borealis of Northern Finland_2017_1013, Biobank of Eastern Finland 1186/2018, Finnish Clinical Biobank Tampere MH0004, Central Finland Biobank 1-2017 and Terveystalo Biobank STB 2018001. The EstBB study was approved by the Ethics Review Committee on Human Research of the University of Tartu. At recruitment, participants signed an informed consent to allow follow-up linkage of their electronic health records. UKBB has approval from the North West Multi-center Research Ethics Committee as a Research Tissue Bank approval. All participants provided an informed consent for their participation in UKBB.

### Reporting summary

Further information on research design is available in the Nature Portfolio Reporting Summary linked to this article.

## Online content

Any methods, additional references, Nature Portfolio reporting summaries, source data, extended data, supplementary information, acknowledgements, peer review information; details of author contributions and competing interests; and statements of data and code availability are available at 10.1038/s41591-022-02122-5.

### Supplementary information

Supplementary Information Supplementary Figs. 1–7 and a list of FinnGen contributors.

Reporting Summary

Supplementary Tables 1−28 Supplementary Tables 1−28.

## Figures and Tables

**Extended Data Table 1:**
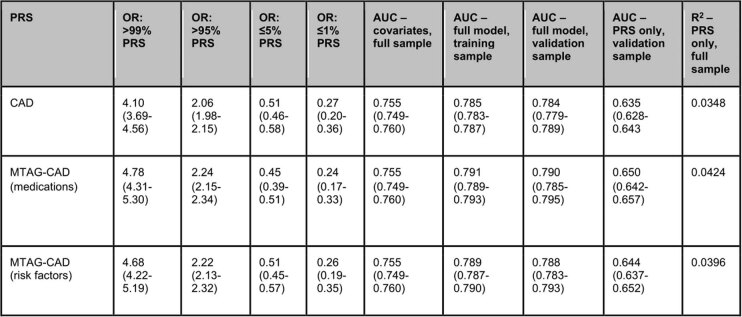
Comparison of associations between the traditional and MTAG-based coronary artery disease (CAD) polygenic risk scores and CAD in the UKBB. The UKBB contained 343,676 samples (17,986 CAD cases). The middle quintile of the PRS (40th to 60th percentiles) was used as the reference group. The AUCs and Nagelkerke pseudo-R^2^ were calculated using PRSs as continuous variables in the models including the PRS and all covariates or only the PRS. 75% of the full sample was used for training and 25% for validation

## Data Availability

The FinnGen GWAS associations for medication-use patterns can be explored with the PheWeb portal (https://med.finngen.fi). The FinnGen release 5 GWAS results for the clinical endpoints can be browsed with the FinnGen PheWeb portal (https://r5.finngen.fi/). Registered researchers can access the medication-use pattern GWAS summary statistics, which have been added to be part of FinnGen R5 public release (https://www.finngen.fi/en/access_results). The FinnGen data may be accessed by approved researchers through Finnish Biobanks’ FinBB portal (www.finbb.fi; email: info.fingenious@finbb.fi). Access to EstBB (https://genomics.ut.ee/en/content/estonian-biobank) and access to UKBB (http://www.ukbiobank.ac.uk/resources/) is restricted for approved researchers and can be requested. Previously reported GWAS associations can be accessed at the NHGRI-EBI GWAS Catalog (https://www.ebi.ac.uk/gwas/) and gnomAD can be accessed at https://gnomad.broadinstitute.org/.

## Code availability

The full genotyping and imputation protocol for FinnGen is described at 10.17504/protocols.io.xbgfijw. Codes for FinnGen pipelines can be accessed at https://github.com/FINNGEN. Codes for ATC- and BNF-based medication phenotypers for the quantitative phenotypes and code (based on the qqman package: https://cran.r-project.org/web/packages/qqman/index.html) to draw a Manhattan plot with added functionality to highlight two sets of SNPs with two different colors can be accessed at https://github.com/yuj1r0/Medication-Use-Patterns.
