# Supplementary material for: Genetic predictors of lifelong medication-use patterns in cardiometabolic diseases
Source: Nat Med. 2023 Jan 18;29(1):209–18. doi: 10.1038/s41591-022-02122-5 (PMC9873570; doi:10.1038/s41591-022-02122-5)
Supplement: Supplementary file 1 — Supplementary Figs. 1–7 and a list of FinnGen contributors. [file 41591_2022_2122_MOESM1_ESM.pdf]

nature medicine

Article

https://doi.org/10.1038/s41591-022-02122-5

Genetic predictors of lifelong medication-use patterns in cardiometabolic diseases

In the format provided by the authors and unedited

Supporting Information

Table of Contents

Supplementary Figure 1 - Genetic components of each FinnGen sample	2
Supplementary Figure 2 - The European and Finnish 1000G genotypes	2
Supplementary Figure 3 - The distribution of kinship values of the FinnGen Study samples	3
Supplementary Figure 4 - Population covariates for 218,957 FinnGen samples	3
Supplementary Figure 5 - Density plots for the year of the recruitment in FinnGen	4
Supplementary Figure 6 - Medication purchases before discontinuation	5
Supplementary Figure 7 - The raw and the transformed quantitative phenotypes	6
FinnGen Contributors	7

Supplementary Figure 1. The scatter plots of the genetic components of each FinnGen sample projected onto the first three principal components

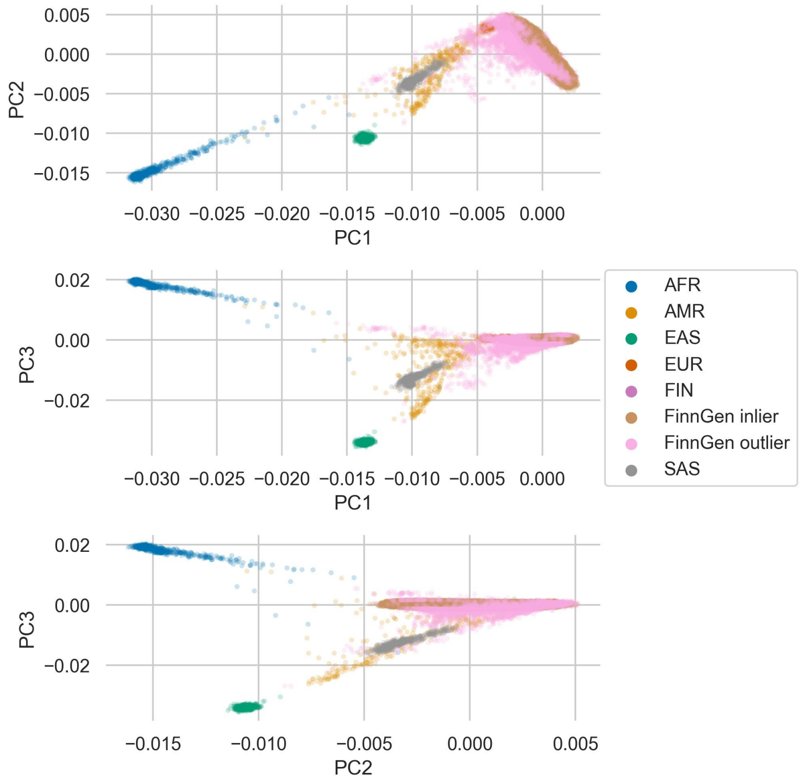

Supplementary Figure 2. The European and Finnish 1000 genomes projected onto the new three-dimensional (principal components 1-3) space

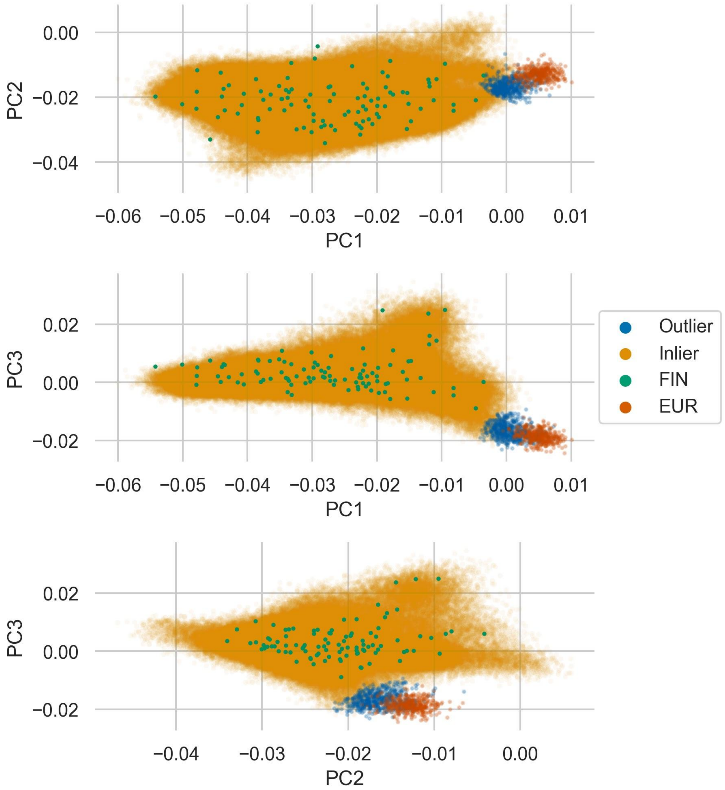

Supplementary Figure 3. The distribution of kinship values of the FinnGen Study samples

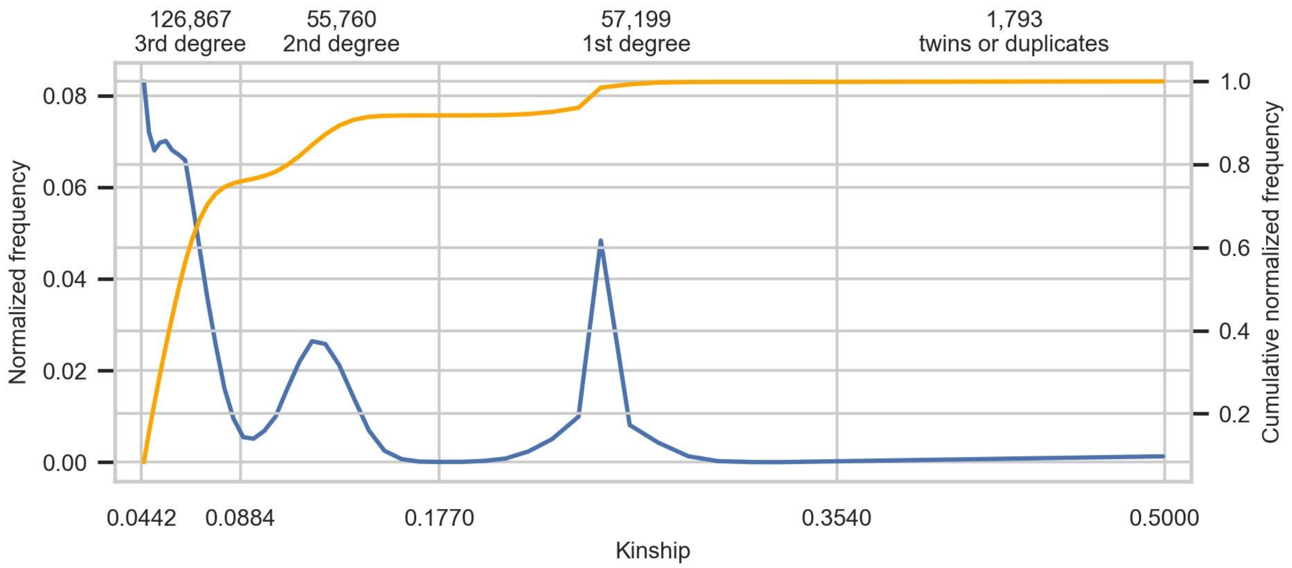

Supplementary Figure 4. Population covariates for 218,957 FinnGen samples from a principal components (PC) analysis performed for the 156,977 unrelated samples after which the 61,980 samples in the second set were projected onto the same multidimensional space, the first 3 PCs are shown

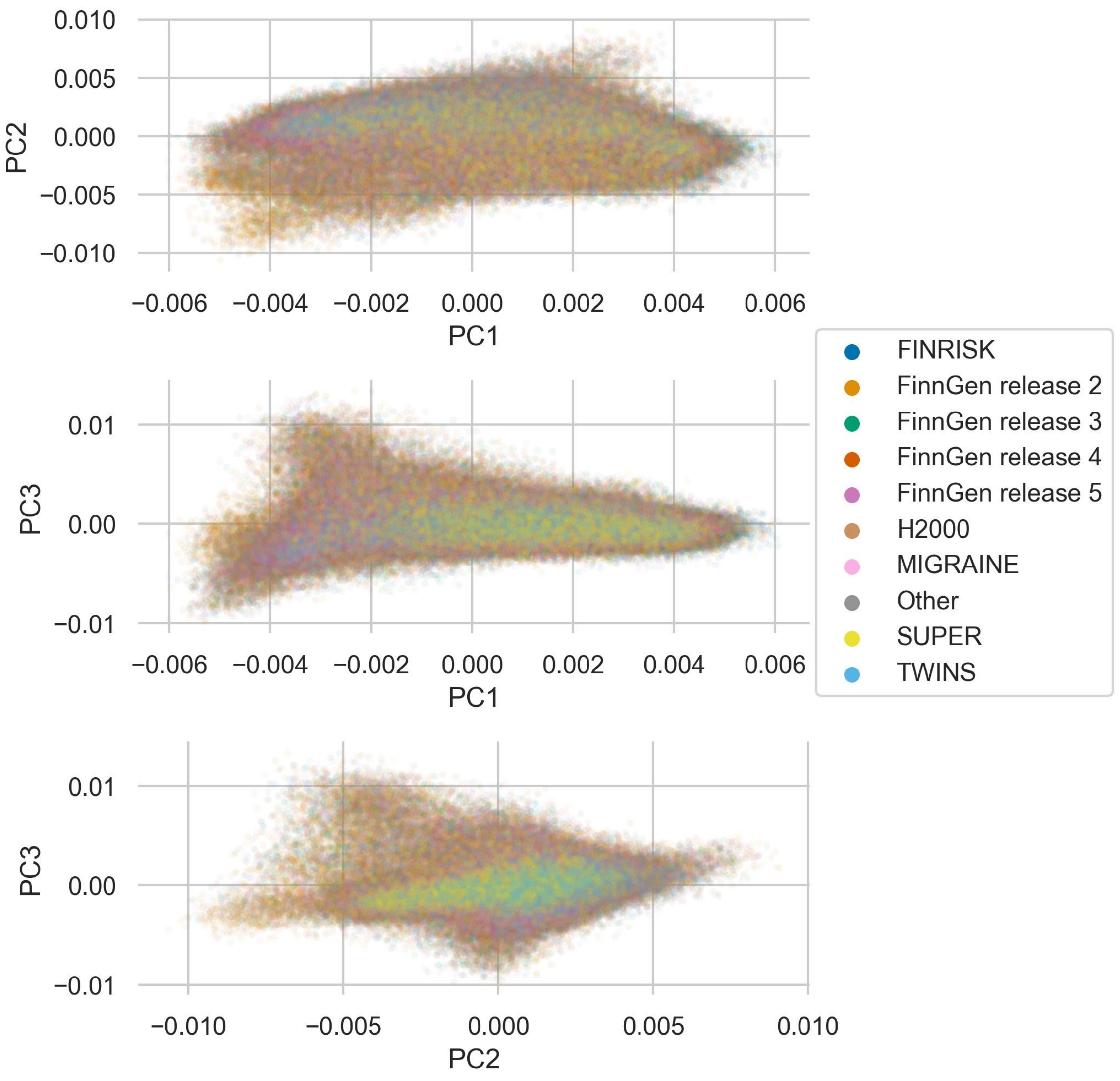

Supplementary Figure 5. Density plots for the year of the recruitment in FinnGen. A significant proportion of the FinnGen R5 participants (n=218,792) are from legacy cohorts collected prior to the FinnGen study and have thus been recruited prior to the official FinnGen study launch in autumn 2017. A) Density plot. B) Cumulative density plot.

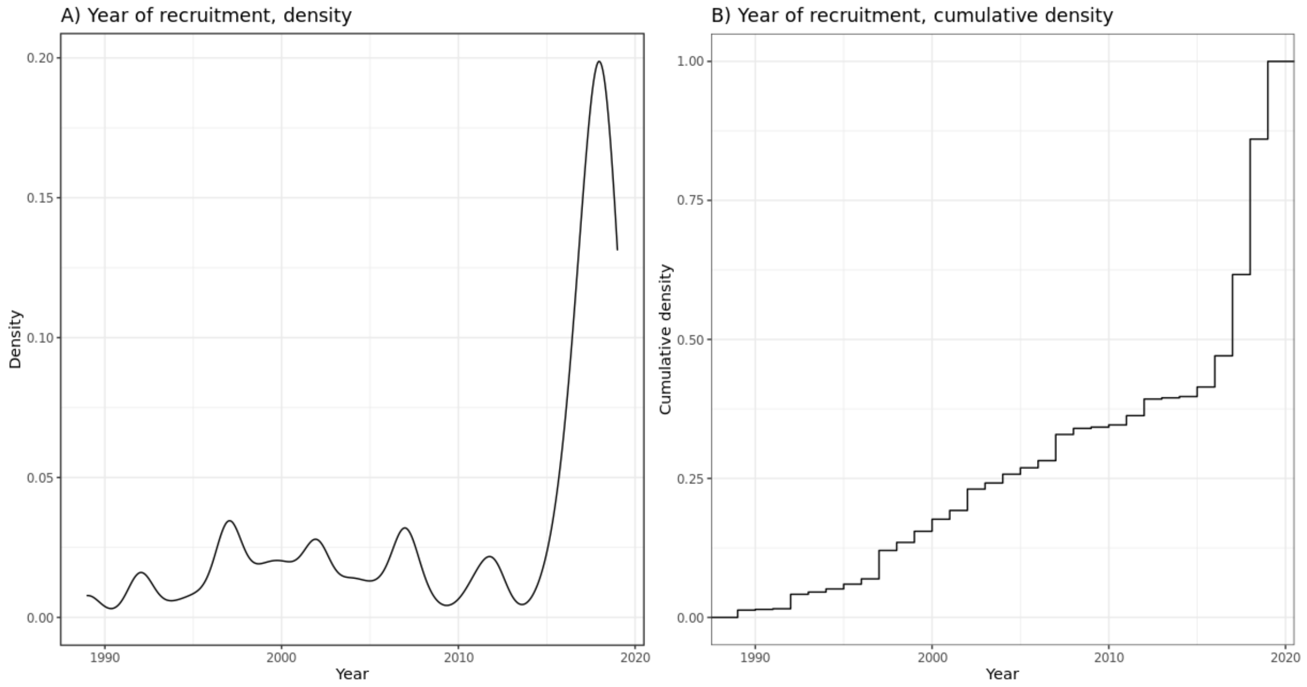

Supplementary Figure 6. Medication purchases before discontinuation. Histograms display the number of individuals for each number of medication purchases before medication discontinuation. Discontinuation here has been defined as having recorded the last purchase at least 1 year before the end of follow-up.

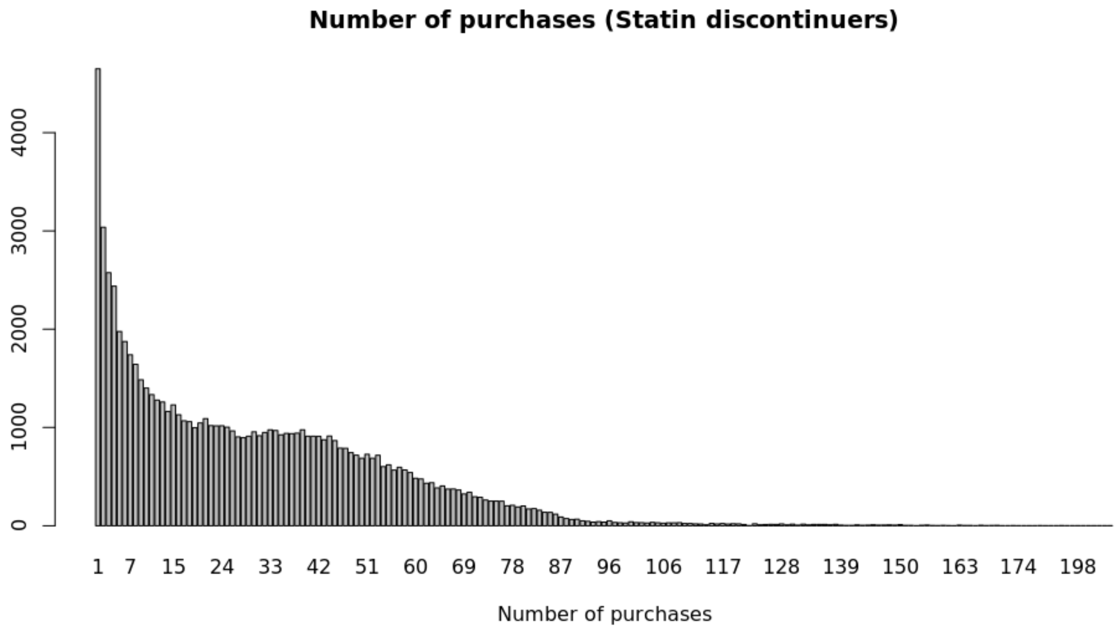

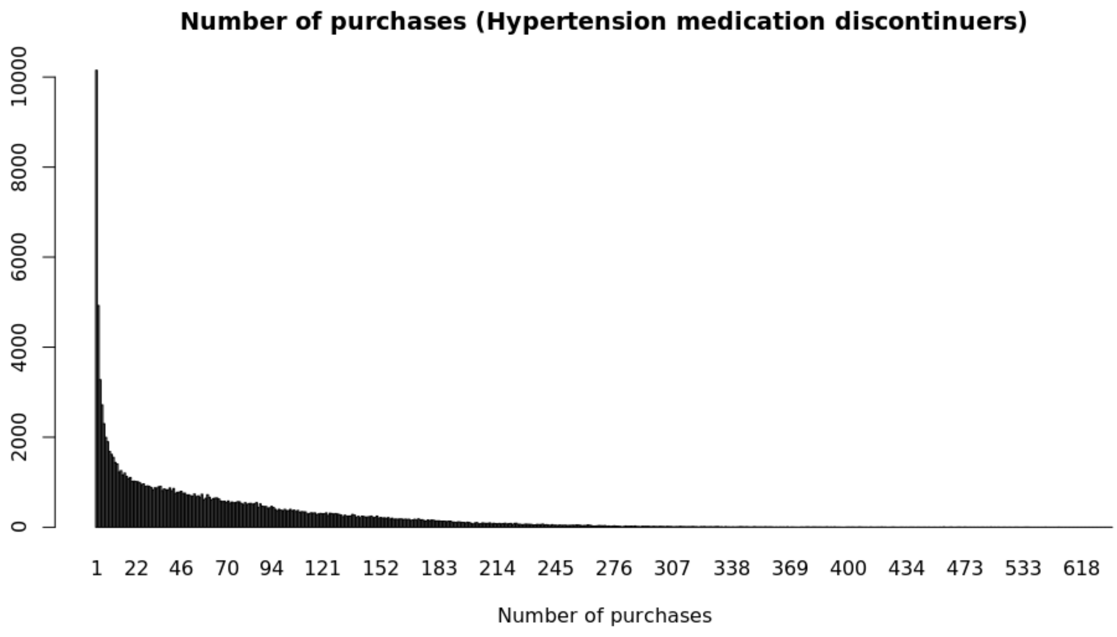

Supplementary Figure 7. The raw and the transformed quantitative phenotypes. Histograms displaying the densities of the raw (A, C, D) and the rank-based inverse normal transformed phenotypes for the total number of purchases of medications used in hyperlipidemia (A-B), hypertension (B-D), and type 2 diabetes (E-F).

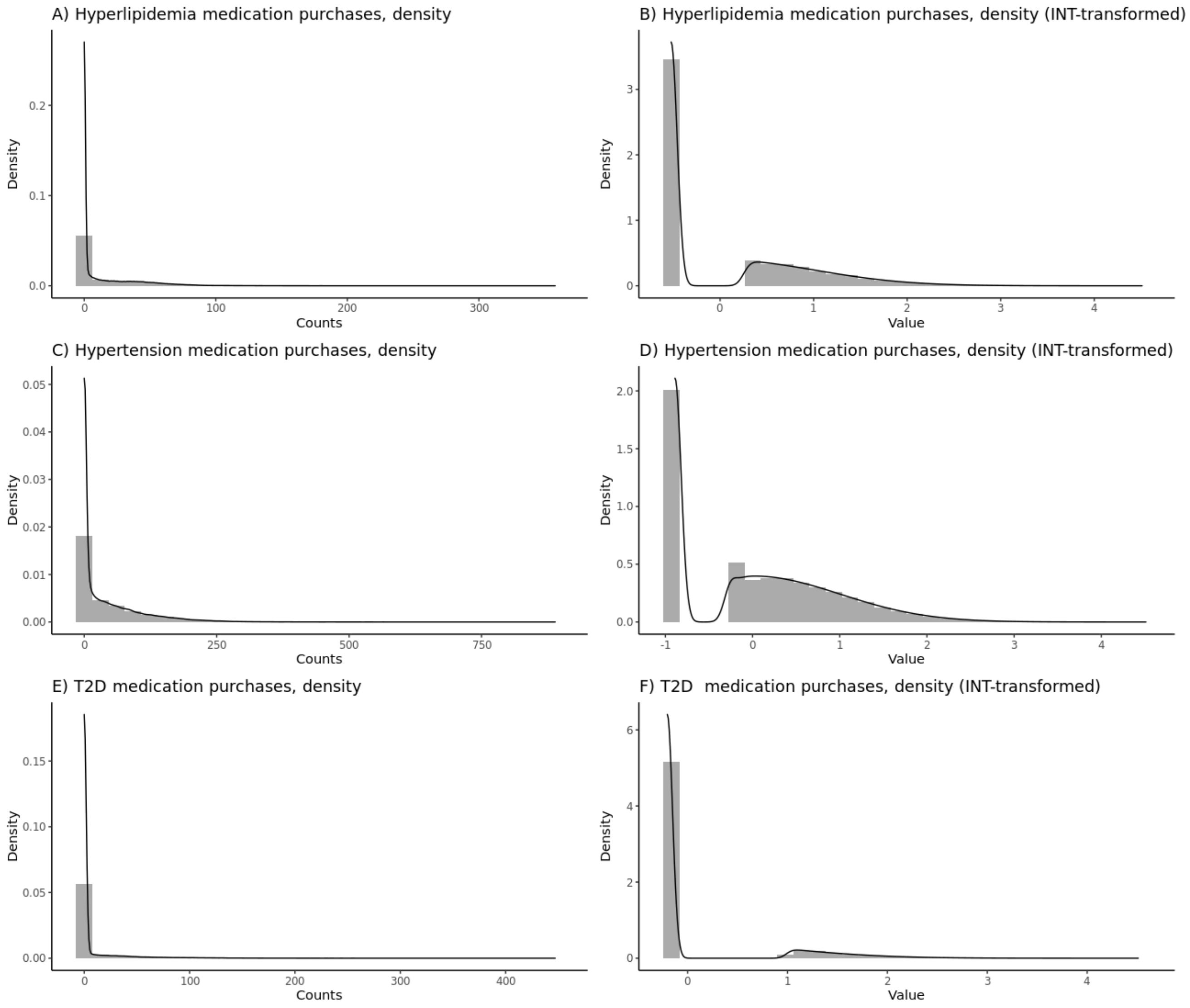

FinnGen Contributors

Steering Committee

Aarno Palotie	Institute for Molecular Medicine Finland (FIMM), HiLIFE, University of Helsinki, Helsinki, Finland; [illegible] Institute of MIT and Harvard; Massachusetts General Hospital
Mark Daly	Institute for Molecular Medicine Finland (FIMM), HiLIFE, University of Helsinki, Helsinki, Finland; Broad Institute of MIT and Harvard; Massachusetts General Hospital

Pharmaceutical Companies

Bridget Riley-Gills	Abbvie, Chicago, IL, United States
Howard Jacob	Abbvie, Chicago, IL, United States
Dirk Paul	Astra Zeneca, Cambridge, United Kingdom
Athena Matakidou	Astra Zeneca, Cambridge, United Kingdom
Adam Platt	Astra Zeneca, Cambridge, United Kingdom
Heiko Runz	Biogen, Cambridge, MA, United States
Sally John	Biogen, Cambridge, MA, United States
George Okafo	Boehringer Ingelheim, Ingelheim am Rhein, Germany
Nathan Lawless	Boehringer Ingelheim, Ingelheim am Rhein, Germany
Robert Plenge	Bristol Myers Squibb, New York, NY, United States
Joseph Maranville	Bristol Myers Squibb, New York, NY, United States
Mark McCarthy	Genentech, San Francisco, CA, United States
Julie Hunkapiller	Genentech, San Francisco, CA, United States
Margaret G. Ehm	GlaxoSmithKline, Collegeville, PA, United States
Kirsi Auro	GlaxoSmithKline, Espoo, Finland
Simonne Longerich	Merck, Kenilworth, NJ, United States
Caroline Fox	Merck, Kenilworth, NJ, United States
Anders Mälarstig	Pfizer, New York, NY, United States
Katherine Klinger	Translational Sciences, Sanofi R&D, Framingham, MA, USA
Deepak Raipal	Translational Sciences, Sanofi R&D, Framingham, MA, USA
Eric Green	Maze Therapeutics, San Francisco, CA, United States
Robert Graham	Maze Therapeutics, San Francisco, CA, United States
Robert Yang	Janssen Biotech, Beerse, Belgium
Chris O´Donnell	Novartis Institutes for BioMedical Research, Cambridge, MA, United States

University of Helsinki & Biobanks

Tomi Mäkelä	HiLIFE, University of Helsinki, Finland, Finland
Jaakko Kaprio	Institute for Molecular Medicine Finland (FIMM), HiLIFE, University of Helsinki, Helsinki, Finland
Petri Virolainen	Auria Biobank / University of Turku / Hospital District of Southwest Finland, Turku, Finland
Antti Hakanen	Auria Biobank / University of Turku / Hospital District of Southwest Finland, Turku, Finland
Terhi Kilpi	THL Biobank / Finnish Institute for Health and Welfare (THL), Helsinki, Finland
Markus Perola	THL Biobank / Finnish Institute for Health and Welfare (THL), Helsinki, Finland
Jukka Partanen	Finnish Red Cross Blood Service / Finnish Hematology Registry and Clinical Biobank, Helsinki, Finland
Anne Pitkäranta	Helsinki Biobank / Helsinki University and Hospital District of Helsinki and Uusimaa, Helsinki
Juhani Junttila	Northern Finland Biobank Borealis / University of Oulu / Northern Ostrobothnia Hospital District, Oulu, Finland
Raisa Serpi	Northern Finland Biobank Borealis / University of Oulu / Northern Ostrobothnia Hospital District, Oulu, Finland
Tarja Laitinen	Finnish Clinical Biobank Tampere / University of Tampere / Pirkanmaa Hospital District, Tampere, Finland
Veli-Matti Kosma	Biobank of Eastern Finland / University of Eastern Finland / Northern Savo Hospital District, Kuopio, Finland
Jari Laukkanen	Central Finland Biobank / University of Jyväskylä / Central Finland Health Care District, Jyväskylä, Finland
Marco Hautalahti	FINBB - Finnish biobank cooperative

Other Experts/ Non-Voting Members

Outi Tuovila	Business Finland, Helsinki, Finland
Raimo Pakkanen	Business Finland, Helsinki, Finland

Scientific Committee

Pharmaceutical companies

Jeffrey Waring	Abbvie, Chicago, IL, United States
Bridget Riley-Gillis	Abbvie, Chicago, IL, United States
Fedik Rahimov	Abbvie, Chicago, IL, United States
Ioanna Tachmazidou	Astra Zeneca, Cambridge, United Kingdom
Chia-Yen Chen	Biogen, Cambridge, MA, United States
Heiko Runz	Biogen, Cambridge, MA, United States
Zhihao Ding	Boehringer Ingelheim, Ingelheim am Rhein, Germany
Marc Jung	Boehringer Ingelheim, Ingelheim am Rhein, Germany
Shameek Biswas	Bristol Myers Squibb, New York, NY, United States
Rion Pendergrass	Genentech, San Francisco, CA, United States

Julie Hunkapiller	Genentech, San Francisco, CA, United States
Margaret G. Ehm	GlaxoSmithKline, Collegeville, PA, United States
David Pulford	GlaxoSmithKline, Stevenage, United Kingdom
Neha Raghavan	Merck, Kenilworth, NJ, United States
Adriana Huertas-Vazquez	Merck, Kenilworth, NJ, United States
Jae-Hoon Sul	Merck, Kenilworth, NJ, United States
Anders Mälarstig	Pfizer, New York, NY, United States
Xinli Hu	Pfizer, New York, NY, United States
Katherine Klinger	Translational Sciences, Sanofi R&D, Framingham, MA, USA
Robert Graham	Maze Therapeutics, San Francisco, CA, United States
Eric Green	Maze Therapeutics, San Francisco, CA, United States
Sahar Mozaffari	Maze Therapeutics, San Francisco, CA, United States
Dawn Waterworth	Janssen Research & Development, LLC, Spring House, PA, United States
Nicole Renaud	Novartis Institutes for BioMedical Research, Cambridge, MA, United States
Ma´en Obeidat	Novartis Institutes for BioMedical Research, Cambridge, MA, United States

University of Helsinki & Biobanks

Samuli Ripatti	Institute for Molecular Medicine Finland (FIMM), HiLIFE, University of Helsinki, Helsinki, Finland
Johanna Schleutker	Auria Biobank / Univ. of Turku / Hospital District of Southwest Finland, Turku, Finland
Markus Perola	THL Biobank / Finnish Institute for Health and Welfare (THL), Helsinki, Finland
Mikko Arvas	Finnish Red Cross Blood Service / Finnish Hematology Registry and Clinical Biobank, Helsinki, Finland
Olli Carpén	Helsinki Biobank / Helsinki University and Hospital District of Helsinki and Uusimaa, Helsinki
Reetta Hinttala	Northern Finland Biobank Borealis / University of Oulu / Northern Ostrobothnia Hospital District, Oulu, Finland
Johannes Kettunen	Northern Finland Biobank Borealis / University of Oulu / Northern Ostrobothnia Hospital District, Oulu, Finland
Arto Mannermaa	Biobank of Eastern Finland / University of Eastern Finland / Northern Savo Hospital District, Kuopio, Finland
Katriina Aalto-Setälä	Faculty of Medicine and Health Technology, Tampere University, Tampere, Finland
Mika Kähönen	Finnish Clinical Biobank Tampere / University of Tampere / Pirkanmaa Hospital District, Tampere, Finland
Jari Laukkanen	Central Finland Biobank / University of Jyväskylä / Central Finland Health Care District, Jyväskylä, Finland
Johanna Mäkelä	FINBB - Finnish biobank cooperative

Clinical Groups

Neurology Group

Reetta Kälviäinen	Northern Savo Hospital District, Kuopio, Finland
Valtteri Julkunen	Northern Savo Hospital District, Kuopio, Finland
Hilkka Soininen	Northern Savo Hospital District, Kuopio, Finland
Anne Remes	Northern Ostrobothnia Hospital District, Oulu, Finland
Mikko Hiltunen	University of Eastern Finland, Kuopio, Finland
Jukka Peltola	Pirkanmaa Hospital District, Tampere, Finland
Minna Raivio	Hospital District of Helsinki and Uusimaa, Helsinki, Finland
Pentti Tienari	Hospital District of Helsinki and Uusimaa, Helsinki, Finland
Juha Rinne	Hospital District of Southwest Finland, Turku, Finland
Roosa Kallionpää	Hospital District of Southwest Finland, Turku, Finland
Juulia Partanen	Institute for Molecular Medicine Finland, HiLIFE, University of Helsinki, Finland
Ali Abbasi	Abbvie, Chicago, IL, United States
Adam Ziemann	Abbvie, Chicago, IL, United States
Nizar Smaoui	Abbvie, Chicago, IL, United States
Anne Lehtonen	Abbvie, Chicago, IL, United States
Susan Eaton	Biogen, Cambridge, MA, United States
Heiko Runz	Biogen, Cambridge, MA, United States
Sanni Lahdenperä	Biogen, Cambridge, MA, United States
Shameek Biswas	Bristol Myers Squibb, New York, NY, United States
Julie Hunkapiller	Genentech, San Francisco, CA, United States
Natalie Bowers	Genentech, San Francisco, CA, United States
Edmond Teng	Genentech, San Francisco, CA, United States
Rion Pendergrass	Genentech, San Francisco, CA, United States
Fanli Xu	GlaxoSmithKline, Brentford, United Kingdom
David Pulford	GlaxoSmithKline, Stevenage, United Kingdom
Kirsi Auro	GlaxoSmithKline, Espoo, Finland
Laura Addis	GlaxoSmithKline, Brentford, United Kingdom
John Eicher	GlaxoSmithKline, Brentford, United Kingdom
Qingqin S Li	Janssen Research & Development, LLC, Titusville, NJ 08560, United States
Karen He	Janssen Research & Development, LLC, Spring House, PA, United States
Ekaterina Khramtsova	Janssen Research & Development, LLC, Spring House, PA, United States

Neha Raghavan	Merck, Kenilworth, NJ, United States

Gastroenterology Group

Martti Färkkilä	Hospital District of Helsinki and Uusimaa, Helsinki, Finland
Jukka Koskela	Hospital District of Helsinki and Uusimaa, Helsinki, Finland
Sampsa Pikkarainen	Hospital District of Helsinki and Uusimaa, Helsinki, Finland
Airi Jussila	Pirkanmaa Hospital District, Tampere, Finland
Katri Kaukinen	Pirkanmaa Hospital District, Tampere, Finland
Timo Blomster	Northern Ostrobothnia Hospital District, Oulu, Finland
Mikko Kiviniemi	Northern Savo Hospital District, Kuopio, Finland
Markku Voutilainen	Hospital District of Southwest Finland, Turku, Finland
Mark Daly	Institute for Molecular Medicine, Finland (FIMM), HiLIFE, University of Helsinki, Helsinki, Finland; Broad Institute of MIT and Harvard; Massachusetts General Hospital
Ali Abbasi	Abbvie, Chicago, IL, United States
Jeffrey Waring	Abbvie, Chicago, IL, United States
Nizar Smaoui	Abbvie, Chicago, IL, United States
Fedik Rahimov	Abbvie, Chicago, IL, United States
Anne Lehtonen	Abbvie, Chicago, IL, United States
Tim Lu	Genentech, San Francisco, CA, United States
Natalie Bowers	Genentech, San Francisco, CA, United States
Rion Pendergrass	Genentech, San Francisco, CA, United States
Linda McCarthy	GlaxoSmithKline, Brentford, United Kingdom
Amy Hart	Janssen Research & Development, LLC, Spring House, PA, United States
Meijian Guan	Janssen Research & Development, LLC, Spring House, PA, United States
Jason Miller	Merck, Kenilworth, NJ, United States
Kirsi Kalpala	Pfizer, New York, NY, United States
Melissa Miller	Pfizer, New York, NY, United States
Xinli Hu	Pfizer, New York, NY, United States

Rheumatology Group

Kari Eklund	Hospital District of Helsinki and Uusimaa, Helsinki, Finland
Antti Palomäki	Hospital District of Southwest Finland, Turku, Finland
Pia Isomäki	Pirkanmaa Hospital District, Tampere, Finland
Laura Pirilä	Hospital District of Southwest Finland, Turku, Finland
Oili Kaipiainen-Seppänen	Northern Savo Hospital District, Kuopio, Finland

Johanna Huhtakangas	Northern Ostrobothnia Hospital District, Oulu, Finland
Nina Mars	Institute for Molecular Medicine Finland (FIMM), HiLIFE, University of Helsinki, Helsinki, Finland
Ali Abbasi	Abbvie, Chicago, IL, United States
Jeffrey Waring	Abbvie, Chicago, IL, United States
Fedik Rahimov	Abbvie, Chicago, IL, United States
Apinya Lertratanakul	Abbvie, Chicago, IL, United States
Nizar Smaoui	Abbvie, Chicago, IL, United States
Anne Lehtonen	Abbvie, Chicago, IL, United States
David Close	Astra Zeneca, Cambridge, United Kingdom
Marla Hochfeld	Bristol Myers Squibb, New York, NY, United States
Natalie Bowers	Genentech, San Francisco, CA, United States
Rion Pendergrass	Genentech, San Francisco, CA, United States
Jorge Esparza Gordillo	GlaxoSmithKline, Brentford, United Kingdom
Kirsi Auro	GlaxoSmithKline, Espoo, Finland
Dawn Waterworth	Janssen Research & Development, LLC, Spring House, PA, United States
Fabiana Farias	Merck, Kenilworth, NJ, United States
Kirsi Kalpala	Pfizer, New York, NY, United States
Nan Bing	Pfizer, New York, NY, United States
Xinli Hu	Pfizer, New York, NY, United States

Pulmonology Group

Tarja Laitinen	Pirkanmaa Hospital District, Tampere, Finland
Margit Pelkonen	Northern Savo Hospital District, Kuopio, Finland
Paula Kauppi	Hospital District of Helsinki and Uusimaa, Helsinki, Finland
Hannu Kankaanranta	University of Gothenburg, Gothenburg, Sweden/ Seinäjoki Central Hospital, Seinäjoki, Finland/ Tampere University, Tampere, Finland
Terttu Harju	Northern Ostrobothnia Hospital District, Oulu, Finland
Riitta Lahesmaa	Hospital District of Southwest Finland, Turku, Finland
Nizar Smaoui	Abbvie, Chicago, IL, United States
Alex Mackay	Astra Zeneca, Cambridge, United Kingdom
Glenda Lassi	Astra Zeneca, Cambridge, United Kingdom
Susan Eaton	Biogen, Cambridge, MA, United States
Hubert Chen	Genentech, San Francisco, CA, United States
Rion Pendergrass	Genentech, San Francisco, CA, United States
Natalie Bowers	Genentech, San Francisco, CA, United States

Joanna Betts	GlaxoSmithKline, Brentford, United Kingdom
Kirsi Auro	GlaxoSmithKline, Espoo, Finland
Rajashree Mishra	GlaxoSmithKline, Brentford, United Kingdom
Majd Mouded	Novartis, Basel, Switzerland
Debby Ngo	Novartis, Basel, Switzerland

Cardiometabolic Diseases Group

Teemu Niiranen	Finnish Institute for Health and Welfare (THL), Helsinki, Finland
Felix Vaura	Finnish Institute for Health and Welfare (THL), Helsinki, Finland
Veikko Salomaa	Finnish Institute for Health and Welfare (THL), Helsinki, Finland
Kaj Metsärinne	Hospital District of Southwest Finland, Turku, Finland
Jenni Aittokallio	Hospital District of Southwest Finland, Turku, Finland
Mika Kähönen	Pirkanmaa Hospital District, Tampere, Finland
Jussi Hernesniemi	Pirkanmaa Hospital District, Tampere, Finland
Daniel Gordin	Hospital District of Helsinki and Uusimaa, Helsinki, Finland
Juha Sinisalo	Hospital District of Helsinki and Uusimaa, Helsinki, Finland
Marja-Riitta Taskinen	Hospital District of Helsinki and Uusimaa, Helsinki, Finland
Tiinamaija Tuomi	Hospital District of Helsinki and Uusimaa, Helsinki, Finland
Timo Hiltunen	Hospital District of Helsinki and Uusimaa, Helsinki, Finland
Jari Laukkanen	Central Finland Health Care District, Jyväskylä, Finland
Amanda Elliott	Institute for Molecular Medicine Finland (FIMM), HiLIFE, University of Helsinki, Helsinki, Finland; Broad Institute, Cambridge, MA, USA and Massachusetts General Hospital, Boston, MA, USA
Mary Pat Reeve	Institute for Molecular Medicine Finland (FIMM), HiLIFE, University of Helsinki, Helsinki, Finland
Sanni Ruotsalainen	Institute for Molecular Medicine Finland (FIMM), HiLIFE, University of Helsinki, Helsinki, Finland
Benjamin Challis	Astra Zeneca, Cambridge, United Kingdom
Dirk Paul	Astra Zeneca, Cambridge, United Kingdom
Julie Hunkapiller	Genentech, San Francisco, CA, United States
Natalie Bowers	Genentech, San Francisco, CA, United States
Rion Pendergrass	Genentech, San Francisco, CA, United States
Audrey Chu	GlaxoSmithKline, Brentford, United Kingdom
Kirsi Auro	GlaxoSmithKline, Espoo, Finland
Dermot Reilly	Janssen Research & Development, LLC, Boston, MA, United States
Mike Mendelson	Novartis, Boston, MA, United States
Jaakko Parkkinen	Pfizer, New York, NY, United States
Melissa Miller	Pfizer, New York, NY, United States

Oncology Group

Tuomo Meretoja	Hospital District of Helsinki and Uusimaa, Helsinki, Finland
Heikki Joensuu	Hospital District of Helsinki and Uusimaa, Helsinki, Finland
Olli Carpén	Hospital District of Helsinki and Uusimaa, Helsinki, Finland
Johanna Mattson	Hospital District of Helsinki and Uusimaa, Helsinki, Finland
Eveliina Salminen	Hospital District of Helsinki and Uusimaa, Helsinki, Finland
Annika Auranen	Pirkanmaa Hospital District , Tampere, Finland
Peeter Karihtala	Northern Ostrobothnia Hospital District, Oulu, Finland
Päivi Auvinen	Northern Savo Hospital District, Kuopio, Finland
Klaus Elenius	Hospital District of Southwest Finland, Turku, Finland
Johanna Schleutker	Hospital District of Southwest Finland, Turku, Finland
Esa Pitkänen	Institute for Molecular Medicine Finland (FIMM), HiLIFE, University of Helsinki, Helsinki, Finland
Nina Mars	Institute for Molecular Medicine Finland (FIMM), HiLIFE, University of Helsinki, Helsinki, Finland
Mark Daly	Institute for Molecular Medicine Finland (FIMM), HiLIFE, University of Helsinki, Helsinki, Finland; Broad Institute of MIT and Harvard; Massachusetts General Hospital
Relja Popovic	Abbvie, Chicago, IL, United States
Jeffrey Waring	Abbvie, Chicago, IL, United States
Bridget Riley-Gillis	Abbvie, Chicago, IL, United States
Anne Lehtonen	Abbvie, Chicago, IL, United States
Jennifer Schutzman	Genentech, San Francisco, CA, United States
Julie Hunkapiller	Genentech, San Francisco, CA, United States
Natalie Bowers	Genentech, San Francisco, CA, United States
Rion Pendergrass	Genentech, San Francisco, CA, United States
Diptee Kulkarni	GlaxoSmithKline, Brentford, United Kingdom
Kirsi Auro	GlaxoSmithKline, Espoo, Finland
Alessandro Porello	Janssen Research & Development, LLC, Spring House, PA, United States
Andrey Loboda	Merck, Kenilworth, NJ, United States
Heli Lehtonen	Pfizer, New York, NY, United States
Stefan McDonough	Pfizer, New York, NY, United States
Sauli Vuoti	Janssen-Cilag Oy, Espoo, Finland

Opthalmology Group

Kai Kaarniranta	Northern Savo Hospital District, Kuopio, Finland
Joni A Turunen	Helsinki University Hospital and University of Helsinki, Helsinki, Finland; Eye Genetics Group,

	Folkhälsan Research Center, Helsinki, Finland
Terhi Ollila	Hospital District of Helsinki and Uusimaa, Helsinki, Finland
Hannu Uusitalo	Pirkanmaa Hospital District, Tampere, Finland
Juha Karjalainen	Institute for Molecular Medicine Finland (FIMM), HiLIFE, University of Helsinki, Helsinki, Finland
Esa Pitkänen	Institute for Molecular Medicine Finland (FIMM), HiLIFE, University of Helsinki, Helsinki, Finland
Mengzhen Liu	Abbvie, Chicago, IL, United States
Heiko Runz	Biogen, Cambridge, MA, United States
Stephanie Loomis	Biogen, Cambridge, MA, United States
Erich Strauss	Genentech, San Francisco, CA, United States
Natalie Bowers	Genentech, San Francisco, CA, United States
Hao Chen	Genentech, San Francisco, CA, United States
Rion Pendergrass	Genentech, San Francisco, CA, United States

Dermatology Group

Kaisa Tasanen	Northern Ostrobothnia Hospital District, Oulu, Finland
Laura Huilaja	Northern Ostrobothnia Hospital District, Oulu, Finland
Katariina Hannula-Jouppi	Hospital District of Helsinki and Uusimaa, Helsinki, Finland
Teea Salmi	Pirkanmaa Hospital District, Tampere, Finland
Sirkku Peltonen	Hospital District of Southwest Finland, Turku, Finland
Leena Koulu	Hospital District of Southwest Finland, Turku, Finland
Nizar Smaoui	Abbvie, Chicago, IL, United States
Fedik Rahimov	Abbvie, Chicago, IL, United States
Anne Lehtonen	Abbvie, Chicago, IL, United States
David Choy	Genentech, San Francisco, CA, United States
Rion Pendergrass	Genentech, San Francisco, CA, United States
Dawn Waterworth	Janssen Research & Development, LLC, Spring House, PA, United States
Kirsi Kalpala	Pfizer, New York, NY, United States
Ying Wu	Pfizer, New York, NY, United States

Odontology Group

Pirkko Pussinen	Hospital District of Helsinki and Uusimaa, Helsinki, Finland
Aino Salminen	Hospital District of Helsinki and Uusimaa, Helsinki, Finland
Tuula Salo	Hospital District of Helsinki and Uusimaa, Helsinki, Finland
David Rice	Hospital District of Helsinki and Uusimaa, Helsinki, Finland
Pekka Nieminen	Hospital District of Helsinki and Uusimaa, Helsinki, Finland

Ulla Palotie	Hospital District of Helsinki and Uusimaa, Helsinki, Finland
Maria Siponen	Northern Savo Hospital District, Kuopio, Finland
Liisa Suominen	Northern Savo Hospital District, Kuopio, Finland
Päivi Mäntylä	Northern Savo Hospital District, Kuopio, Finland
Ulvi Gursoy	Hospital District of Southwest Finland, Turku, Finland
Vuokko Anttonen	Northern Ostrobothnia Hospital District, Oulu, Finland
Kirsi Sipilä	Research Unit of Oral Health Sciences Faculty of Medicine, University of Oulu, Oulu, Finland; Medical Research Center, Oulu, Oulu University Hospital and University of Oulu, Oulu, Finland
Rion Pendergrass	Genentech, San Francisco, CA, United States

Women's Health and Reproduction Group

Hannele Laivuori	Institute for Molecular Medicine Finland (FIMM), HiLIFE, University of Helsinki, Helsinki, Finland
Venla Kurra	Pirkanmaa Hospital District, Tampere, Finland
Laura Kotaniemi-Talonen	Pirkanmaa Hospital District, Tampere, Finland
Oskari Heikinheimo	Hospital District of Helsinki and Uusimaa, Helsinki, Finland
Ilkka Kalliala	Hospital District of Helsinki and Uusimaa, Helsinki, Finland
Lauri Aaltonen	Hospital District of Helsinki and Uusimaa, Helsinki, Finland
Varpu Jokimaa	Hospital District of Southwest Finland, Turku, Finland
Johannes Kettunen	Northern Ostrobothnia Hospital District, Oulu, Finland
Marja Vääräsmäki	Northern Ostrobothnia Hospital District, Oulu, Finland
Outi Uimari	Northern Ostrobothnia Hospital District, Oulu, Finland
Laure Morin-Papunen	Northern Ostrobothnia Hospital District, Oulu, Finland
Maarit Niinimäki	Northern Ostrobothnia Hospital District, Oulu, Finland
Terhi Piltonen	Northern Ostrobothnia Hospital District, Oulu, Finland
Katja Kivinen	Institute for Molecular Medicine Finland (FIMM), HiLIFE, University of Helsinki, Helsinki, Finland
Elisabeth Widen	Institute for Molecular Medicine Finland (FIMM), HiLIFE, University of Helsinki, Helsinki, Finland
Taru Tukiainen	Institute for Molecular Medicine Finland (FIMM), HiLIFE, University of Helsinki, Helsinki, Finland
Mary Pat Reeve	Institute for Molecular Medicine Finland (FIMM), HiLIFE, University of Helsinki, Helsinki, Finland
Mark Daly	Institute for Molecular Medicine Finland (FIMM), HiLIFE, University of Helsinki, Helsinki, Finland; Broad Institute of MIT and Harvard; Massachusetts General Hospital
Niko Välimäki	University of Helsinki, Helsinki, Finland
Eija Laakkonen	University of Jyväskylä, Jyväskylä, Finland
Jaakko Tyrmi	University of Oulu, Oulu, Finland / University of Tampere, Tampere, Finland
Heidi Silven	University of Oulu, Oulu, Finland
Eeva Sliz	University of Oulu, Oulu, Finland
Riikka Arffman	University of Oulu, Oulu, Finland

Susanna Savukoski	University of Oulu, Oulu, Finland
Triin Laisk	Estonian biobank, Tartu, Estonia
Natalia Pujol	Estonian biobank, Tartu, Estonia
Mengzhen Liu	Abbvie, Chicago, IL, United States
Bridget Riley-Gillis	Abbvie, Chicago, IL, United States
Rion Pendergrass	Genentech, San Francisco, CA, United States
Janet Kumar	GlaxoSmithKline, Collegeville, PA, United States
Kirsi Auro	GlaxoSmithKline, Espoo, Finland

Depression Group

Iiris Hovatta	University of Helsinki, Finland
Chia-Yen Chen	Biogen, Cambridge, MA, United States
Erkki Isometsä	Hospital District of Helsinki and Uusimaa, Helsinki, Finland
Kumar Veerapen	Broad Institute, Cambridge, MA, United States
Hanna Ollila	Institute for Molecular Medicine Finland (FIMM), HiLIFE, University of Helsinki, Helsinki, Finland
Jaana Suvisaari	Finnish Institute for Health and Welfare (THL), Helsinki, Finland
Thomas Damm Als	Aarhus University, Denmark

ENT (ear, nose and throat) Group

Antti Mäkitie	Department of Otorhinolaryngology - Head and Neck Surgery, University of Helsinki and Helsinki University Hospital, Helsinki, Finland
Argyro Bizaki-Vallaskangas	Pirkanmaa Hospital District, Tampere, Finland
Sanna Toppila-Salmi	University of Helsinki, Finland
Tytti Willberg	Hospital District of Southwest Finland, Turku, Finland
Elmo Saarentaus	Institute for Molecular Medicine Finland (FIMM), HiLIFE, University of Helsinki, Helsinki, Finland
Antti Aarnisalo	Hospital District of Helsinki and Uusimaa, Helsinki, Finland
Eveliina Salminen	Hospital District of Helsinki and Uusimaa, Helsinki, Finland
Elisa Rahikkala	Northern Ostrobothnia Hospital District, Oulu, Finland
Johannes Kettunen	Northern Ostrobothnia Hospital District, Oulu, Finland

POI (premature ovarian failure) Group

Kristiina Aittomäki	Department of Medical Genetics, Helsinki University Central Hospital, Helsinki, Finland

LiverScore Group

Fredrik Åberg	Transplantation and Liver Surgery Clinic, Helsinki University Hospital, Helsinki University, Helsinki,

Finland

FinnGen Analysis Working Group

Mitja Kurki	Institute for Molecular Medicine Finland (FIMM), HiLIFE, University of Helsinki, Helsinki, Finland; Broad Institute, Cambridge, MA, United States
Samuli Ripatti	Institute for Molecular Medicine Finland (FIMM), HiLIFE, University of Helsinki, Helsinki, Finland
Mark Daly	Institute for Molecular Medicine, Finland (FIMM), HiLIFE, University of Helsinki, Helsinki, Finland; Broad Institute of MIT and Harvard; Massachusetts General Hospital
Juha Karjalainen	Institute for Molecular Medicine Finland (FIMM), HiLIFE, University of Helsinki, Helsinki, Finland
Aki Havulinna	Institute for Molecular Medicine Finland (FIMM), HiLIFE, University of Helsinki, Helsinki, Finland; Finnish Institute for Health and Welfare (THL), Helsinki, Finland
Juha Mehtonen	Institute for Molecular Medicine Finland (FIMM), HiLIFE, University of Helsinki, Helsinki, Finland
Priit Palta	Institute for Molecular Medicine Finland (FIMM), HiLIFE, University of Helsinki, Helsinki, Finland
Shabbeer Hassan	Institute for Molecular Medicine Finland (FIMM), HiLIFE, University of Helsinki, Helsinki, Finland
Pietro Della Briotta Parolo	Institute for Molecular Medicine Finland (FIMM), HiLIFE, University of Helsinki, Helsinki, Finland
Wei Zhou	Broad Institute, Cambridge, MA, United States
Mutaamba Maasha	Broad Institute, Cambridge, MA, United States
Kumar Veerapen	Broad Institute, Cambridge, MA, United States
Shabbeer Hassan	Institute for Molecular Medicine Finland (FIMM), HiLIFE, University of Helsinki, Helsinki, Finland
Susanna Lemmelä	Institute for Molecular Medicine Finland (FIMM), HiLIFE, University of Helsinki, Helsinki, Finland
Manuel Rivas	University of Stanford, Stanford, CA, United States
Mari E. Niemi	Institute for Molecular Medicine Finland (FIMM), HiLIFE, University of Helsinki, Helsinki, Finland
Aarno Palotie	Institute for Molecular Medicine Finland (FIMM), HiLIFE, University of Helsinki, Helsinki, Finland
Aoxing Liu	Institute for Molecular Medicine Finland (FIMM), HiLIFE, University of Helsinki, Helsinki, Finland
Arto Lehisto	Institute for Molecular Medicine Finland (FIMM), HiLIFE, University of Helsinki, Helsinki, Finland
Andrea Ganna	Institute for Molecular Medicine Finland (FIMM), HiLIFE, University of Helsinki, Helsinki, Finland
Vincent Llorens	Institute for Molecular Medicine Finland (FIMM), HiLIFE, University of Helsinki, Helsinki, Finland
Hannele Laivuori	Institute for Molecular Medicine Finland (FIMM), HiLIFE, University of Helsinki, Helsinki, Finland
Taru Tukiainen	Institute for Molecular Medicine Finland (FIMM), HiLIFE, University of Helsinki, Helsinki, Finland
Mary Pat Reeve	Institute for Molecular Medicine Finland (FIMM), HiLIFE, University of Helsinki, Helsinki, Finland
Henrike Heyne	Institute for Molecular Medicine Finland (FIMM), HiLIFE, University of Helsinki, Helsinki, Finland
Nina Mars	Institute for Molecular Medicine Finland (FIMM), HiLIFE, University of Helsinki, Helsinki, Finland
Joel Rämö	Institute for Molecular Medicine Finland (FIMM), HiLIFE, University of Helsinki, Helsinki, Finland
Elmo Saarentaus	Institute for Molecular Medicine Finland (FIMM), HiLIFE, University of Helsinki, Helsinki, Finland
Hanna Ollila	Institute for Molecular Medicine Finland (FIMM), HiLIFE, University of Helsinki, Helsinki, Finland

Rodos Rodosthenous	Institute for Molecular Medicine Finland (FIMM), HiLIFE, University of Helsinki, Helsinki, Finland
Satu Strausz	Institute for Molecular Medicine Finland (FIMM), HiLIFE, University of Helsinki, Helsinki, Finland
Tuula Palotie	University of Helsinki and Hospital District of Helsinki and Uusimaa, Helsinki, Finland
Kimmo Palin	University of Helsinki, Helsinki, Finland
Javier Garcia-Tabuenca	University of Tampere, Tampere, Finland
Harri Siirtola	University of Tampere, Tampere, Finland
Tuomo Kiiskinen	Institute for Molecular Medicine Finland (FIMM), HiLIFE, University of Helsinki, Helsinki, Finland
Jiwoo Lee	Institute for Molecular Medicine Finland (FIMM), HiLIFE, University of Helsinki, Helsinki, Finland; Broad Institute, Cambridge, MA, United States
Kristin Tsuo	Institute for Molecular Medicine Finland (FIMM), HiLIFE, University of Helsinki, Helsinki, Finland; Broad Institute, Cambridge, MA, United States
Amanda Elliott	Institute for Molecular Medicine Finland (FIMM), HiLIFE, University of Helsinki, Helsinki, Finland; Broad Institute, Cambridge, MA, USA and Massachusetts General Hospital, Boston, MA, USA
Kati Kristiansson	THL Biobank / Finnish Institute for Health and Welfare (THL), Helsinki, Finland
Mikko Arvas	Finnish Red Cross Blood Service / Finnish Hematology Registry and Clinical Biobank, Helsinki, Finland
Kati Hyvärinen	Finnish Red Cross Blood Service, Helsinki, Finland
Jarmo Ritari	Finnish Red Cross Blood Service, Helsinki, Finland
Olli Carpén	Helsinki Biobank / Helsinki University and Hospital District of Helsinki and Uusimaa, Helsinki
Johannes Kettunen	Northern Finland Biobank Borealis / University of Oulu / Northern Ostrobothnia Hospital District, Oulu, Finland
Katri Pylkäs	University of Oulu, Oulu, Finland
Eeva Sliz	University of Oulu, Oulu, Finland
Minna Karjalainen	University of Oulu, Oulu, Finland
Tuomo Mantere	Northern Finland Biobank Borealis / University of Oulu / Northern Ostrobothnia Hospital District, Oulu, Finland
Eeva Kangasniemi	Finnish Clinical Biobank Tampere / University of Tampere / Pirkanmaa Hospital District, Tampere, Finland
Sami Heikkinen	University of Eastern Finland, Kuopio, Finland
Arto Mannermaa	Biobank of Eastern Finland / University of Eastern Finland / Northern Savo Hospital District, Kuopio, Finland
Eija Laakkonen	University of Jyväskylä, Jyväskylä, Finland
Nina Pitkänen	Auria Biobank / University of Turku / Hospital District of Southwest Finland, Turku, Finland
Samuel Lessard	Translational Sciences, Sanofi R&D, Framingham, MA, USA
Clément Chatelain	Translational Sciences, Sanofi R&D, Framingham, MA, USA

Biobank directors

Perttu Terho	Auria Biobank / University of Turku / Hospital District of Southwest Finland, Turku, Finland
Sirpa Soini	THL Biobank / Finnish Institute for Health and Welfare (THL), Helsinki, Finland
Jukka Partanen	Finnish Red Cross Blood Service / Finnish Hematology Registry and Clinical Biobank, Helsinki, Finland
Eero Punkka	Helsinki Biobank / Helsinki University and Hospital District of Helsinki and Uusimaa, Helsinki
Raisa Serpi	Northern Finland Biobank Borealis / University of Oulu / Northern Ostrobothnia Hospital District, Oulu, Finland
Sanna Siltanen	Finnish Clinical Biobank Tampere / University of Tampere / Pirkanmaa Hospital District, Tampere, Finland
Veli-Matti Kosma	Biobank of Eastern Finland / University of Eastern Finland / Northern Savo Hospital District, Kuopio, Finland
Teijo Kuopio	Central Finland Biobank / University of Jyväskylä / Central Finland Health Care District, Jyväskylä, Finland

FinnGen Teams

Administration

Anu Jalanko	Institute for Molecular Medicine Finland (FIMM), HiLIFE, University of Helsinki, Helsinki, Finland
Huei-Yi Shen	Institute for Molecular Medicine Finland (FIMM), HiLIFE, University of Helsinki, Helsinki, Finland
Risto Kajanne	Institute for Molecular Medicine Finland (FIMM), HiLIFE, University of Helsinki, Helsinki, Finland
Mervi Aavikko	Institute for Molecular Medicine Finland (FIMM), HiLIFE, University of Helsinki, Helsinki, Finland

Analysis

Mitja Kurki	Institute for Molecular Medicine Finland (FIMM), HiLIFE, University of Helsinki, Helsinki, Finland; Broad Institute, Cambridge, MA, United States
Juha Karjalainen	Institute for Molecular Medicine Finland (FIMM), HiLIFE, University of Helsinki, Helsinki, Finland
Pietro Della Briotta Parolo	Institute for Molecular Medicine Finland (FIMM), HiLIFE, University of Helsinki, Helsinki, Finland
Arto Lehisto	Institute for Molecular Medicine Finland (FIMM), HiLIFE, University of Helsinki, Helsinki, Finland
Juha Mehtonen	Institute for Molecular Medicine Finland (FIMM), HiLIFE, University of Helsinki, Helsinki, Finland
Wei Zhou	Broad Institute, Cambridge, MA, United States
Masahiro Kanai	Broad Institute, Cambridge, MA, United States
Mutaamba Maasha	Broad Institute, Cambridge, MA, United States
Kumar Veerapen	Broad Institute, Cambridge, MA, United States

Clinical Endpoint Development

Hannele Laivuori	Institute for Molecular Medicine Finland (FIMM), HiLIFE, University of Helsinki, Helsinki, Finland

Aki Havulinna	Institute for Molecular Medicine Finland (FIMM), HiLIFE, University of Helsinki, Helsinki, Finland; Finnish Institute for Health and Welfare (THL), Helsinki, Finland
Susanna Lemmelä	Institute for Molecular Medicine Finland (FIMM), HiLIFE, University of Helsinki, Helsinki, Finland
Tuomo Kiiskinen	Institute for Molecular Medicine Finland (FIMM), HiLIFE, University of Helsinki, Helsinki, Finland
L. Elisa Lahtela	Institute for Molecular Medicine Finland (FIMM), HiLIFE, University of Helsinki, Helsinki, Finland

Communication

Mari Kaunisto	Institute for Molecular Medicine Finland (FIMM), HiLIFE, University of Helsinki, Helsinki, Finland

E-Science

Elina Kilpeläinen	Institute for Molecular Medicine Finland (FIMM), HiLIFE, University of Helsinki, Helsinki, Finland
Timo P. Sipilä	Institute for Molecular Medicine Finland (FIMM), HiLIFE, University of Helsinki, Helsinki, Finland
Oluwaseun Alexander Dada	Institute for Molecular Medicine Finland (FIMM), HiLIFE, University of Helsinki, Helsinki, Finland
Awaisa Ghazal	Institute for Molecular Medicine Finland (FIMM), HiLIFE, University of Helsinki, Helsinki, Finland
Anastasia Kytölä	Institute for Molecular Medicine Finland (FIMM), HiLIFE, University of Helsinki, Helsinki, Finland
Rigbe Weldatsadik	Institute for Molecular Medicine Finland (FIMM), HiLIFE, University of Helsinki, Helsinki, Finland

Genotyping

Kati Donner	Institute for Molecular Medicine Finland (FIMM), HiLIFE, University of Helsinki, Helsinki, Finland
Timo P. Sipilä	Institute for Molecular Medicine Finland (FIMM), HiLIFE, University of Helsinki, Helsinki, Finland

Sample Collection Coordination

Anu Loukola	Helsinki Biobank / Helsinki University and Hospital District of Helsinki and Uusimaa, Helsinki

Sample Logistics

Päivi Laiho	THL Biobank / Finnish Institute for Health and Welfare (THL), Helsinki, Finland
Tuuli Sistonen	THL Biobank / Finnish Institute for Health and Welfare (THL), Helsinki, Finland
Essi Kaiharju	THL Biobank / Finnish Institute for Health and Welfare (THL), Helsinki, Finland
Markku Laukkanen	THL Biobank / Finnish Institute for Health and Welfare (THL), Helsinki, Finland
Elina Järvensivu	THL Biobank / Finnish Institute for Health and Welfare (THL), Helsinki, Finland
Sini Lähteenmäki	THL Biobank / Finnish Institute for Health and Welfare (THL), Helsinki, Finland
Lotta Männikkö	THL Biobank / Finnish Institute for Health and Welfare (THL), Helsinki, Finland
Regis Wong	THL Biobank / Finnish Institute for Health and Welfare (THL), Helsinki, Finland
Auli Toivola	THL Biobank / Finnish Institute for Health and Welfare (THL), Helsinki, Finland

Registry Data Operations

Minna Brunfeldt	THL Biobank / Finnish Institute for Health and Welfare (THL), Helsinki, Finland
Hannele Mattsson	THL Biobank / Finnish Institute for Health and Welfare (THL), Helsinki, Finland
Kati Kristiansson	THL Biobank / Finnish Institute for Health and Welfare (THL), Helsinki, Finland
Susanna Lemmelä	Institute for Molecular Medicine Finland (FIMM), HiLIFE, University of Helsinki, Helsinki, Finland
Sami Koskelainen	THL Biobank / Finnish Institute for Health and Welfare (THL), Helsinki, Finland
Tero Hiekkalinna	THL Biobank / Finnish Institute for Health and Welfare (THL), Helsinki, Finland
Teemu Paajanen	THL Biobank / Finnish Institute for Health and Welfare (THL), Helsinki, Finland

Sequencing Informatics

Priit Palta	Institute for Molecular Medicine Finland (FIMM), HiLIFE, University of Helsinki, Helsinki, Finland
Kalle Pärn	Institute for Molecular Medicine Finland (FIMM), HiLIFE, University of Helsinki, Helsinki, Finland
Mart Kals	Institute for Molecular Medicine Finland (FIMM), HiLIFE, University of Helsinki, Helsinki, Finland
Shuang Luo	Institute for Molecular Medicine Finland (FIMM), HiLIFE, University of Helsinki, Helsinki, Finland
Vishal Sinha	Institute for Molecular Medicine Finland (FIMM), HiLIFE, University of Helsinki, Helsinki, Finland

Trajectory

Tarja Laitinen	Pirkanmaa Hospital District, Tampere, Finland
Mary Pat Reeve	Institute for Molecular Medicine Finland (FIMM), HiLIFE, University of Helsinki, Helsinki, Finland
Marianna Niemi	University of Tampere, Tampere, Finland
Kumar Veerapen	Broad Institute, Cambridge, MA, United States
Harri Siirtola	University of Tampere, Tampere, Finland
Javier Gracia-Tabuenca	University of Tampere, Tampere, Finland
Mika Helminen	University of Tampere, Tampere, Finland
Tiina Luukkaala	University of Tampere, Tampere, Finland
Iida Vähätalo	University of Tampere, Tampere, Finland

Data protection officer

Jyrki Pitkänen	Institute for Molecular Medicine Finland (FIMM), HiLIFE, University of Helsinki, Helsinki, Finland

FINBB - Finnish biobank cooperative

Marco Hautalahti	Finnish Biobank Cooperative - FINBB

Johanna Mäkelä	Finnish Biobank Cooperative - FINBB
Sarah Smith	Finnish Biobank Cooperative - FINBB
Tom Southerington	Finnish Biobank Cooperative - FINBB
