# Supplementary material for: Genetic predictors of lifelong medication-use patterns in cardiometabolic diseases
Source: Nat Med. 2023 Jan 18;29(1):209–18. doi: 10.1038/s41591-022-02122-5 (PMC9873570; doi:10.1038/s41591-022-02122-5)
Supplement: Supplementary file 2 — Reporting Summary [file 41591_2022_2122_MOESM2_ESM.pdf]

nature portfolio

Corresponding author(s): Samuli Ripatti

Last updated by author(s): Nov 5, 2022

Reporting Summary

Nature Portfolio wishes to improve the reproducibility of the work that we publish. This form provides structure for consistency and transparency in reporting. For further information on Nature Portfolio policies, see our Editorial Policies and the Editorial Policy Checklist.

Statistics

For all statistical analyses, confirm that the following items are present in the figure legend, table legend, main text, or Methods section.

n/a	Confirmed	
☐	☒	The exact sample size (*n*) for each experimental group/condition, given as a discrete number and unit of measurement
☐	☒	A statement on whether measurements were taken from distinct samples or whether the same sample was measured repeatedly
☐	☒	The statistical test(s) used AND whether they are one- or two-sided<br>*Only common tests should be described solely by name; describe more complex techniques in the Methods section.*
☐	☒	A description of all covariates tested
☐	☒	A description of any assumptions or corrections, such as tests of normality and adjustment for multiple comparisons
☐	☒	A full description of the statistical parameters including central tendency (e.g. means) or other basic estimates (e.g. regression coefficient) AND variation (e.g. standard deviation) or associated estimates of uncertainty (e.g. confidence intervals)
☐	☒	For null hypothesis testing, the test statistic (e.g. *F*, *t*, *r*) with confidence intervals, effect sizes, degrees of freedom and *P* value noted<br>*Give P values as exact values whenever suitable.*
☐	☒	For Bayesian analysis, information on the choice of priors and Markov chain Monte Carlo settings
☐	☒	For hierarchical and complex designs, identification of the appropriate level for tests and full reporting of outcomes
☐	☒	Estimates of effect sizes (e.g. Cohen's *d*, Pearson's *r*), indicating how they were calculated

Our web collection on statistics for biologists contains articles on many of the points above.

Software and code

Policy information about availability of computer code

Data collection

For this study, no software was used for data collection.

Data analysis

Software: R versions v3.6.0, v4.0.2, v4.0.3 and v4.1.2 (https://www.r-project.org/), PLINK v2.0, Eagle v2.3, AxiomGT1 algorithm for Affymetrix data (Thermo Fisher Scientific, Santa Clara, CA, USA), Beagle v.28Sep18.793, PRS-CS (https://github.com/getian107/PRScs), IMPUTE4, SISu v3, SAIGE (version 0.35.8.8, 0.43.1, https://github.com/weizhouUMICH/SAIGE), LDSC v1.0.1 (https://github.com/bulik/ldsc), GWAMA v2.2.2

Codes for FinnGen pipelines can be accessed at https://github.com/FINNGEN. Analysis pipelines: FineMapping pipeline (https://github.com/FINNGEN/finemapping-pipeline), genetic ancestry and PCA pipeline (https://github.com/FINNGEN/pca_kinship), PRS-CS pipeline (https://github.com/FINNGEN/CS-PRS-pipeline). The full genotyping and imputation protocol for FinnGen is described at https://doi.org/10.17504/protocols.io.xbgfijw.

Codes for ATC- and BNF-based medication phenotypers for the quantitative phenotypes and code (based on the qqman package: https://cran.r-project.org/web/packages/qqman/index.html) to draw a Manhattan plot with added functionality to highlight two sets of SNPs with two different colors can be accessed at https://github.com/yuj1r0/Medication-Use-Patterns.

For manuscripts utilizing custom algorithms or software that are central to the research but not yet described in published literature, software must be made available to editors and reviewers. We strongly encourage code deposition in a community repository (e.g. GitHub). See the Nature Portfolio guidelines for submitting code & software for further information.

Data

Policy information about availability of data

All manuscripts must include a data availability statement. This statement should provide the following information, where applicable:

- Accession codes, unique identifiers, or web links for publicly available datasets
- A description of any restrictions on data availability
- For clinical datasets or third party data, please ensure that the statement adheres to our policy

The FinnGen GWAS associations for medication use patterns can be explored with the PheWeb portal (https://med.finngen.fi). The summary statistics have been added to be part of FinnGen R5 public release (https://www.finngen.fi/en/access_results). The FinnGen release 5 GWAS results for the clinical endpoints can be browsed with the FinnGen PheWeb portal (https://r5.finngen.fi/). The FinnGen data may be accessed through Finnish Biobanks' FinBB portal (web link: www.finbb.fi, email: info.fingenious@finbb.fi). Access to Estonian Biobank (https://genomics.ut.ee/en/content/estonian-biobank) and access to UK Biobank (http://www.ukbiobank.ac.uk/resources/) can be requested. Previously reported GWAS associations can be accessed at The NHGRI-EBI GWAS Catalog (https://www.ebi.ac.uk/gwas/) and gnomAD can be accessed (https://gnomad.broadinstitute.org/).

Human research participants

Policy information about studies involving human research participants and Sex and Gender in Research.

Reporting on sex and gender	Sex was imputed with PLINK. All participants were included in the analyses regardless of sex, and Imputed sex was included as a covariate in all analyses, excluding sex-stratified analyses. Effect sizes of genome-wide significant loci lead variants were compared in sex-stratified analyses.
Population characteristics	Our main dataset, FinnGen release 5, consisted of 218,792 genotyped individuals of Finnish ancestry with a total of 5,118,565 years of drug-registry-based follow-up. Of the cohort 56.5% were female and the mean age at the end of the follow-up was 59.8 years. A total of 44,343,661 drug purchases were recorded and 3,650,495 (8.2%) of these were drugs used in the treatment of hyperlipidemia, hypertension, or T2D. The Estonian Biobank (EstBB) consisted of 184,892 individuals (females: 65.5%, mean age: 44.2 years) and the UK Biobank (UKBB) consisted of 343, 676 individuals (females: 53.7%, mean age: 56.9 years).
Recruitment	Data in FinnGen Data Freeze 5 are administred by regional biobanks (Auria Biobank, Biobank of Central Finland, Biobank of Eastern Finland, Biobank of Eastern Finland, Borealis Biobank, Helsinki Biobank, Tampere Biobank), the Blood Service Biobank, the Terveystalo Biobank, and biobanks administered by the Finnish Institute for Health and Welfare. EstBB is a population-based cohort of 200,000 participants. At recruitment, participants have signed a consent to allow follow-up linkage of their electronic health records. UKBB comprises phenotype data from 500,000 volunteer individuals from the UK population aged between 40 and 69 during recruitment in 2006-2010. Data for all participants have been linked with national Hospital Episode statistics.
Ethics oversight	The FinnGen Study protocol UHUS/990/2017) was approved by the Ethics Review Board of the Hospital District of Helsinki and Uusimaa. EstBB study was approved by the Ethics Review Committee on Human Research of the University of Tartu. UKBB has approval from the North West Multi-centre Research Ethics Committee (MREC) as a Research Tissue Bank (RTB) approval.

Note that full information on the approval of the study protocol must also be provided in the manuscript.

Field-specific reporting

Please select the one below that is the best fit for your research. If you are not sure, read the appropriate sections before making your selection.

☒ Life sciences ☐ Behavioural & social sciences ☐ Ecological, evolutionary & environmental sciences

For a reference copy of the document with all sections, see nature.com/documents/nr-reporting-summary-flat.pdf

Life sciences study design

All studies must disclose on these points even when the disclosure is negative.

Sample size	Sample sizes were determined by the current biobank sample sizes of FinnGen, EstBB and UKBB. The sample sizes were considered sufficient as performing genome-wide association studies in biobanks of these sizes (hundreds of thousands), including meta-analysis, is the state-of-the-art for genomic discovery for polygenic traits.
Data exclusions	In FinnGen and EstBB we excluded participants dead or less than 10 years at the beginning of the follow-up from the quantitative analyses (not expected to have any prescription medication purchases). In UKBB the analyses were restricted to "white British" ethnicity (self-reported, UKBB Data-field 21000), and participants without any prescriptions were excluded from the GWAS analyses.

Replication	For the GWAS analyses, quantitative phenotypes were analyzed in 3 biobanks: FinnGen, EstBB and UKBB. Binary GWAS analyses were performed in FinnGen and EstBB. A high concordance of effect directions (87.2%) was checked between the biobanks for all 303 significant (p<5e-8) loci lead variants in FinnGen. A meta-analysis was performed for all loci with a suggestive association (p<5e-6) in FinnGen: quantitative traits (FinnGen, EstBB, UKBB) and binary traits (FinnGen + EstBB). 333 loci were significant (p<5e-9), including 94 loci not significant (p≥5e-8) in the initial FinnGen analyses. Of the all 347 CS containing fine-mapped loci-phenotype associations (loci lead variant p<5e-8 and ≥1 95% CS) in FinnGen, 282 (81.3%) were genome-wine significant in the meta-analysis (p<5e-9).
Randomization	Analyses were performed in population based biobanks. Participants were allocated into groups by their medication use behavior (quantitative and binary phenotypes). Age, Age2, follow-up time (end of the follow-up – 1.1.1995), sex (imputed with PLINK, not included in the sex-stratified analyses), the first 10 first principal components of ancestry, and genotyping batch (for batches with at least 10 cases and controls) were used as covariates.
Blinding	Blinding was not relevant as no intervention occured. The data points were collected from the biobank participants medical records and blood samples (genotype data), and all data were collected before any of the analyzes of this study were planned (so this study protocol could not have had any effect on any data points). Also, blinding of the studied predictors (genomes), was not applicable. The data was pseudonymized, and the participants could not be identified nor interacted with.

Reporting for specific materials, systems and methods

We require information from authors about some types of materials, experimental systems and methods used in many studies. Here, indicate whether each material, system or method listed is relevant to your study. If you are not sure if a list item applies to your research, read the appropriate section before selecting a response.

Materials & experimental systems

n/a	Involved in the study
☒	☐ Antibodies
☒	☐ Eukaryotic cell lines
☒	☐ Palaeontology and archaeology
☒	☐ Animals and other organisms
☒	☐ Clinical data
☒	☐ Dual use research of concern

Methods

n/a	Involved in the study
☒	☐ ChIP-seq
☒	☐ Flow cytometry
☒	☐ MRI-based neuroimaging
